# MYSM1 inhibits human colorectal cancer tumorigenesis by activating miR-200 family members/CDH1 and blocking PI3K/AKT signaling

**DOI:** 10.1186/s13046-021-02106-2

**Published:** 2021-10-27

**Authors:** Xu Chen, Wei Wang, Yufang Li, Yi Huo, Han Zhang, Fan Feng, Wenjin Xi, Tianze Zhang, Jinjian Gao, Fan Yang, Siyi Chen, Angang Yang, Tao Wang

**Affiliations:** 1 State Key Laboratory of Cancer Biology, Department of Immunology, Fourth Military Medical University, Xi’an, Shaanxi 710032 P.R. China; 2 Air Force Health Care Center for Special Services, Hangzhou, Zhejiang 310007 P.R. China; 3 Nuclear Medicine Diagnostic Center, Shaanxi Provincial People’s Hospital, Xi’an, Shaanxi 710032 P.R. China; 4 Department of Medical Genetics and Developmental Biology, Fourth Military Medical University, Xi’an, Shaanxi 710032 P.R. China; 5 Department of Digestive Surgery, Xijing Hospital of Digestive Diseases, Fourth Military Medical University, Xi’an, Shaanxi 710032 P.R. China; 6 Department of Molecular Microbiology and Immunology, Norris Comprehensive Cancer Center, Keck School of Medicine, University of Southern California, Los Angeles, CA USA

**Keywords:** MYSM1, Colorectal cancer, EMT, miR-200, PI3K/AKT

## Abstract

**Background:**

Histone epigenetic modification disorder is an important predisposing factor for the occurrence and development of many cancers, including colorectal cancer (CRC). The role of MYSM1, a metalloprotease that deubiquitinates monoubiquitinated histone H2A, in colorectal cancer was identified to evaluate its potential clinical application value.

**Methods:**

MYSM1 expression levels in CRC cell lines and tumor tissues were detected, and their associations with patient survival rate and clinical stage were analyzed using databases and tissue microarrays. Gain- and loss-of-function studies were performed to identify the roles of MYSM1 in CRC cell proliferation, apoptosis, cell cycle progression, epithelial-mesenchymal transition (EMT) and metastasis in vitro and in vivo. ChIP, rescue assays and signal pathway verification were conducted for mechanistic study. Immunohistochemistry (IHC) was used to further assess the relationship of MYSM1 with CRC diagnosis and prognosis.

**Results:**

MYSM1 was significantly downregulated and was related to the overall survival (OS) of CRC patients. MYSM1 served as a CRC suppressor by inducing apoptosis and inhibiting cell proliferation, EMT, tumorigenic potential and metastasis. Mechanistically, MYSM1 directly bound to the promoter region of miR-200/CDH1, impaired the enrichment of repressive H2AK119ub1 modification and epigenetically enhanced miR-200/CDH1 expression. Testing of paired CRC patient samples confirmed the positive regulatory relationship between MYSM1 and miR-200/CDH1. Furthermore, silencing MYSM1 stimulated PI3K/AKT signaling and promoted EMT in CRC cells. More importantly, a positive association existed between MYSM1 expression and a favorable CRC prognosis.

**Conclusions:**

MYSM1 plays essential suppressive roles in CRC tumorigenesis and is a potential target for reducing CRC progression and distant metastasis.

**Supplementary Information:**

The online version contains supplementary material available at 10.1186/s13046-021-02106-2.

## Background

Ubiquitination is one of the main histone posttranscriptional modifications that regulate chromatin structure and affect DNA biological processes such as gene transcription and DNA damage repair. Importantly, the cellular machinery regulating histone ubiquitination is frequently altered in cancers. Specifically, numerous immunohistochemical analyses have revealed that aberrant histone ubiquitination patterns exist in many cancer types [1–3]. In agreement with these observations, genes encoding histone E3 ubiquitin ligases and deubiquitinases (DUBs) are also frequently altered in cancers [4], and many of the enzymes possess tumor suppressor potential (e.g., BAP1, USP16 and RNF20) or oncogenic potential (e.g., BMI1 and USP22) [5–8].

Histone H2A was the first protein identified to be modified by ubiquitin and is the most abundant ubiquitinated protein in the nucleus [9]. K119 is the most frequently observed histone H2A ubiquitination site, and monoubiquitination of K119 (H2AK119ub1) occurs on approximately 10% of all nucleosomal H2As [9]. The predominant E3 ubiquitin ligase that catalyzes H2AK119ub1 is the catalytic subunit of the polycomb repressive complex (PRC1), which is composed of RING1A and RING1B and activated by BMI1 [10], while BAP1, USP16 and Myb-like SWIRM and MPN domain 1 (MYSM1) are the main H2AK119ub1 DUBs [3, 11, 12]. H2AK119ub1 is enriched within promoter regions of polycomb target genes and functions as a transcriptional repressor through a variety of mechanisms [13]. H2AK119ub1-mediated repression of polycomb target genes is necessary for the maintenance of stem cell populations, and dynamic regulation of H2AK119ub1 controls both normal hematopoiesis and maintenance of cancer stem cells [11, 14, 15]. For example, BMI1 is overexpressed and promotes cancer cell self-renewal in multiple cancer types, including acute myeloid leukemia, glioblastoma multiforme (GBM), colorectal cancer (CRC), and epithelial ovarian cancer [14, 16–19]. In contrast, reduced expression of BAP1 occurs frequently in metastatic uveal melanoma, pleural mesothelioma, and clear-cell renal cell carcinoma [5, 20–22]. Collectively, the existing studies strongly suggest that high H2AK119ub1 levels are associated with tumorigenesis; thus, H2AK119ub1 has become a potential target for tumor therapy [23].

MYSM1, which is also known as 2A-DUB, removes monoubiquitin from H2AK119ub1 and collaborates with histone acetylation to activate transcription [3]. Early studies found that MYSM1 mainly regulates the homeostasis of hematopoietic stem cells (HSCs) and the differentiation and functions of immune cells, including B cells, T cells, dendritic cells (DCs) and natural killer (NK) cells [24–29]. We previously demonstrated that MYSM1 deletion drives HSCs from quiescence into rapid cycling and increases the HSC apoptotic rate, resulting in exhaustion of the stem cell pool, which leads to impaired self-renewal and lineage reconstitution abilities [26]. Recent studies have shown that MYSM1 has a wide range of functions, and its deficiency not only causes serious inflammatory reactions, anemia and other immune system disorders but also leads to dysfunction of multiple tissues and organs [30–34]. However, the current knowledge regarding the mechanisms underlying the coordination between MYSM1 and tumors remains limited.

In this study, we investigated the role of MYSM1 in CRC and explored its potential mechanisms. Our results show that MYSM1 suppresses CRC progression by coordinating epigenetic regulation of miR-200 family members and CDH1 and by inhibiting PI3K/AKT signaling. Our results suggest that MYSM1 might be a potential therapeutic and prognostic target for CRC.

## Methods

### Human CRC patient specimens

Paraffin-embedded human CRC patient specimens, including paired adjacent normal mucosal tissues (N), primary tumor tissues (T) and lymphatic or hepatic metastasis tissues (M), were obtained from 41 CRC patients (Additional file 1: Table S1) selected from the Digestive Disease Department of Xijing Hospital affiliated with the Fourth Military Medical University, Xi’an, Shaanxi, China. All enrolled patients had been diagnosed with CRC in both primary and metastatic sites. All patients enrolled from 2010 to 2015 had undergone specific surgery for primary and metastatic tumors at the Digestive Disease Department of Xijing Hospital. This research was approved by the Medical Ethics Committee of Fourth Military Medical University, and all patients signed informed consent forms. Careful microdissection was performed.

**Fig. 1:**
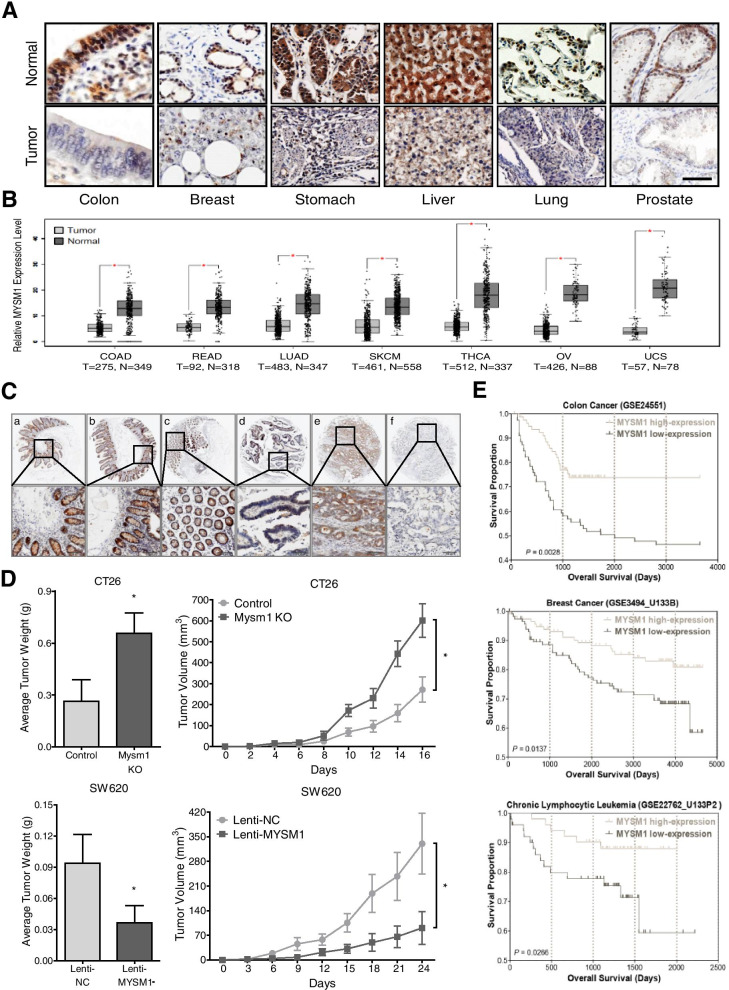
MYSM1 is negatively associated with tumorigenesis in CRC. **A** Representative IHC of MYSM1 expression in different organs using a tissue microarray of normal (*upper*) and tumor (*lower*) tissues. *Scale bars:* 100 μm. **B** Different expression levels of MYSM1 in a series of tumor and paired normal tissues were analyzed based on the GEPIA database. The data are shown as the means ± SDs (*P < 0.05). **C** Representative IHC of MYSM1 expression in stage I-IV (a-f) CRC samples in the tissue microarray. *Scale bars:* 100 μm. **D** Engineered CT26 cells (control: wild-type; Mysm1 KO: Mysm1 knocked out by CRISPR-Cas9) or SW620 cells (Lenti-NC: infected with lentiviral vehicle; Lenti-MYSM1: infected with MYSM1-overexpressing lentivirus) were transplanted subcutaneously into athymic BALB/c mice. The masses and growth curves of the neoplasms are shown. The data are presented as the means ± SDs (*P < 0.05, *n* = 3 independent experiments). **E** The OS in CRC (GSE24551), breast cancer (GSE3494_U133B) and chronic lymphocytic leukemia (CLL) (GSE22762_U133P2) was analyzed with a Kaplan-Meier survival curve. MYSM1 expression data from the GEO database were divided into two groups using the median. Statistical significance was assessed via the *P* log-rank test

**Fig. 2:**
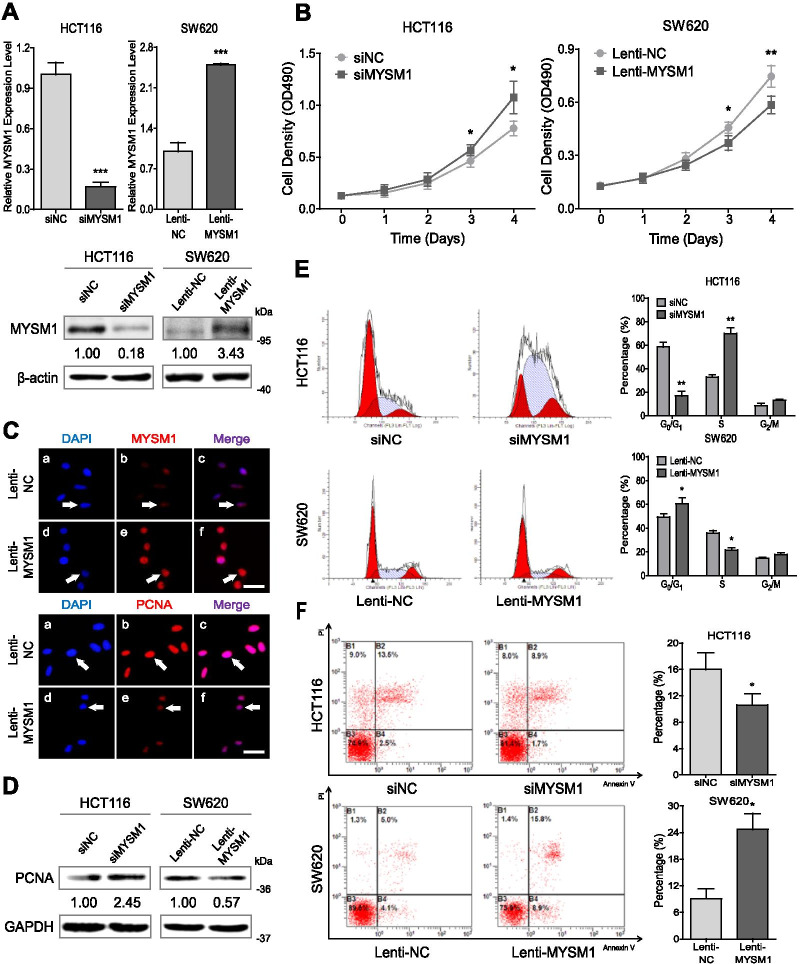
The clinical value of MYSM1 in the diagnosis and prognosis of CRC was evaluated. **A** *Left*: Representative IHC of MYSM1 in 38 adjacent normal (N), 53 adenocarcinoma (T) and 33 lymph node metastatic carcinoma (M) tissues from the same tissue microarray. *Right*: Graphs showing the relative frequencies of the immunoreactivity scores of MYSM1 in 124 samples. *Scale bars:* 200 μm (*low power*), 50 μm (*high power*). **B** *Left*: Scatter plot of the immunoreactivity scores of MYSM1 in CRC samples from 80 adjacent normal tissues (N) and 100 carcinomas (T) in the same tissue microarray. *Right*: Graphs indicating the distribution of the immunoreactivity scores of MYSM1 in 180 samples. The data are shown as the means ± SDs (*P < 0.05). **C** *Left*: Representative IHC of MYSM1 in stage I-IV CRC samples from 98 CRC patients. *Right*: Graphs showing the relative frequencies of the immunoreactivity scores of MYSM1 in different stages of CRC. *Scale bars:* 50 μm. **D** Kaplan-Meier survival curve analysis of the OS of 100 CRC patients from the same tissue microarray. The *P* log-rank test was used to determine statistical significance. **E** *Left*: Representative IHC of MYSM1 in samples of different histological subtypes, including mucinous adenocarcinoma (MA), tubular adenocarcinoma (TA) and adenocarcinoma (A), from 99 CRC patients. *Right*: Graphs indicating the distribution of the immunoreactivity scores of MYSM1 in different histologic subtypes. *Scale bars:* 50 μm. **F** Schematic diagram summarizing our model for studying the suppressive effects of MYSM1 on the EMT process. The immunoreactivity scores were based on the following scale: absent (0), moderate (2–3), elevated (4–5) and strong (6–7)

Three separate tissue microarrays containing normal/tumor tissues from different human organs (Fig. 1A), CRC samples of patients with stage I-IV disease (Fig. 1C), and adjacent normal/tumor/metastasis tissues from CRC patients (Fig. 2A and Additional file 2: Table S2) were purchased from Alenabio (Xi’an, China). Another tissue microarray for the survival study (Fig. 2B-E and Additional file 3: Table S3) was obtained from Outdo Biotech (Shanghai, China).

### Immunohistochemistry (IHC)

For IHC, paraffin-embedded sections of CRC tissues were deparaffinized with xylene and treated with serial dilutions of ethanol. The slides were boiled for 15 min in 10 mM sodium citrate at pH 6.0 for antigen retrieval. Then, endogenous peroxidase activity was blocked with 3% H_2_O_2_ for 10 min, and the sections were blocked for 1 h in blocking solution (5% normal goat serum in phosphate-buffered saline, PBS). The sections were incubated with primary antibodies that diluted in antibody diluent for IHC (Beyotime, Shanghai, China) in a humidified chamber at 4 °C overnight. Then, the sections were incubated with the appropriate secondary antibodies (Maxim, Fuzhou, China) for 30 min before an additional 10 min incubation with streptavidin horseradish peroxidase (HRP, Maxim) at room temperature. Visualization was performed with 3,3′-diaminobenzidine (DAB, Maxim) for 1–2 min, and the sections were counterstained with hematoxylin (Maxim). The sections were dehydrated and mounted in Eukitt medium. A light microscope (Olympus, Shinjuku, Japan) with identical camera settings was used to capture the images.

To balance the intensity and quantity of positive staining during IHC, immunoreactivity scores of staining intensity/quantity were used. Briefly, the percentage (quantity) of positive cells (PCs) was scored as follows: PCs = 0%, score = 0; PCs ≤ 25%, score = 1; 25% < PCs ≤ 50%, score = 2; 50% < PCs ≤ 75%, score = 3; and PCs > 75%, score = 4. In addition, the intensity of staining in PCs was scored as follows: colorless, score = 0; light yellow, score = 1; deep yellow, score = 2; and dark brown, score = 3. Finally, the immunoreactivity score of each sample was determined by adding its quantity score to the intensity score. According to the total score, staining was classified as absent (0), moderate (2–3), elevated (4–5), or strong (6–7). The samples with elevated (4–5) or strong (6–7) expression were identified as IHC-positive.

### Tumor xenografts and metastasis assays in vivo

MYSM1 expression was stably overexpressed via lentiviral particle infection or steadily knocked out with CRISPR-Cas9 in target cells. For in vivo proliferation assays, 5 × 10^6^ cells were harvested, resuspended in 200 μL of serum-free medium and injected subcutaneously into the backs of 6- to 8-week-old female athymic BALB/c mice. Five animals were used in each experiment per group. The neoplasms were examined every 2–3 days with Vernier calipers, and the volume was calculated using the following formula: 1/6 × length × width^2^ [35]. After three or 4 weeks (depending on the tumors), the neoplasms were removed from under the skin of the experimental mice and immediately measured. For the in vivo metastasis assay, 5 × 10^6^ SW620/Lenti-MYSM1 or SW620/Lenti-negative control (NC) cells were suspended in 200 μL of serum-free medium and injected into the tail veins of 6- to 8-week-old female athymic BALB/c mice (five mice per group). After 6 weeks, the mice were sacrificed, and the individual organs, especially the lungs and livers, were removed and observed visually. All animal experiments were approved by the Institutional Animal Care and Use Committee of Fourth Military Medical University. In the proliferation and metastasis assays, any experimental mice that died earlier than the planned endpoint were excluded from the analysis.

### Chromatin immunoprecipitation (ChIP)

A ChIP assay was performed with a Chromatin Immunoprecipitation Assay Kit (Millipore, Billerica, USA) according to the manufacturer’s instructions. Briefly, experimental cells were harvested and crosslinked to DNA with 1% formaldehyde for 15 min. The cell lysates were resuspended in RIPA buffer with a protease inhibitor cocktail (Roche, Indianapolis, USA). Then, the chromatin was sonicated to shear the DNA into 150- to 250-base pair (bp) fragments. The sheared chromatin was incubated with magnetic beads and immunoprecipitating antibodies at 4 °C overnight. The beads containing the immunoprecipitates were washed multiple times, and DNA was recovered as follows: the DNA crosslinks were chelated, the DNA was eluted and incubated with Proteinase K, and the DNA was extracted with phenol-chloroform and precipitated with ethanol. The purified DNA (10 ng/reaction) was quantified by qRT-PCR using different sets of primers to amplify the miR-200b-a-429, miR-200c-141 and CDH1 promoter regions near the transcription start sites (TSSs). The relative fold enrichment of each molecule was normalized to that of the NC (IgG).

### Oligonucleotides, antibodies and primers

Information regarding the CRC patients involved in the clinical studies is provided in Additional files 1, 2, 3: Tables S1, S2, S3. Information regarding the oligonucleotides and antibodies is provided in Additional file 4: Table S4 and Additional file 5: Table S5. Information regarding the primers is provided in Additional files 6, 7, 8, 9: Table S6, S7, S8, S9.

### Other applied methods

The other cell lines used and the cultivation, transfection, lentivirus construction and infection, qRT-PCR, western blot, growth curve assay, colony formation assay, cell cycle and apoptosis analysis, wound healing and Transwell assay, and immunofluorescence (IF) protocols are further described in Additional file 10: Supplementary methods.

### Statistical analysis

The data were analyzed with SPSS 25.0 software and are presented as the means ± standard deviations (SDs) from at least triplicate experiments. The statistical significance of any difference between two independent groups was tested by two-tailed Student’s t-test. More than two groups were compared by using one-way ANOVA with Bonferroni’s multiple comparisons test. The significance of the survival curves was estimated with the Kaplan-Meier method and analyzed by the *P* log-rank test. The significance of the correlations between mRNAs and miRNAs or between mRNAs and mRNAs was evaluated via the Spearman rank correlation test. The differences in the characteristics of the two groups were examined by the chi-squared test and Fisher’s exact test. The diagnostic accuracy (sensitivity and specificity) was assessed according to receiver operating characteristic (ROC) curves via area under the curve (AUC) analysis. The results were considered statistically significant at *P <  0.05, **P <  0.01 and ***P <  0.001.

## Results

### MYSM1 is downregulated and serves as a potential tumor suppressor in CRC

To better investigate the potential correlation between MYSM1 and tumors, we first performed IHC on a series of tumor and normal tissue microarrays to identify variations in the expression level of MYSM1. The results showed that MYSM1 was dominantly expressed in the nucleus in nearly all human tissues and organs (Additional file 11: Figure S1A) but was significantly downregulated in tumors, especially those in the colon, breasts, stomach, liver, lungs and prostate (Fig. 1A). Then, we analyzed bioinformatics data from the Gene Expression Profiling Interactive Analysis (GEPIA) website (http://gepia.cancer-pku.cn) that were mainly from The Cancer Genome Atlas (TCGA) and discovered that the expression of MYSM1 in normal tissues was dramatically higher than that in tumor specimens (Additional file 11: Figure S1B). Notably, MYSM1 expression in adjacent normal tissues derived from 349 colon adenocarcinoma (COAD) and 318 rectal adenocarcinoma (READ) patients was significantly higher than that in 275 COAD and 92 READ specimens. Similar trends were also observed in large samples of lung adenocarcinoma (LUAD), skin cutaneous melanoma (SKCM), thyroid carcinoma (THCA), ovarian serous cystadenocarcinoma (OV) and uterine carcinosarcoma (UCS) tissues, as revealed by scatter diagrams (Fig. 1B), likely indicating a reverse relationship between MYSM1 and tumors. In addition, we found that as the CRC stage gradually increased, MYSM1 expression markedly decreased (Fig. 1C). Interestingly, higher MYSM1 expression levels were detected in epithelial CRC cell lines (HCT116, HT29, SW480 and CACO2) than in mesenchymal CRC cell lines (SW620 and LOVO) (Additional file 11: Figure S1C).

**Fig. 3:**
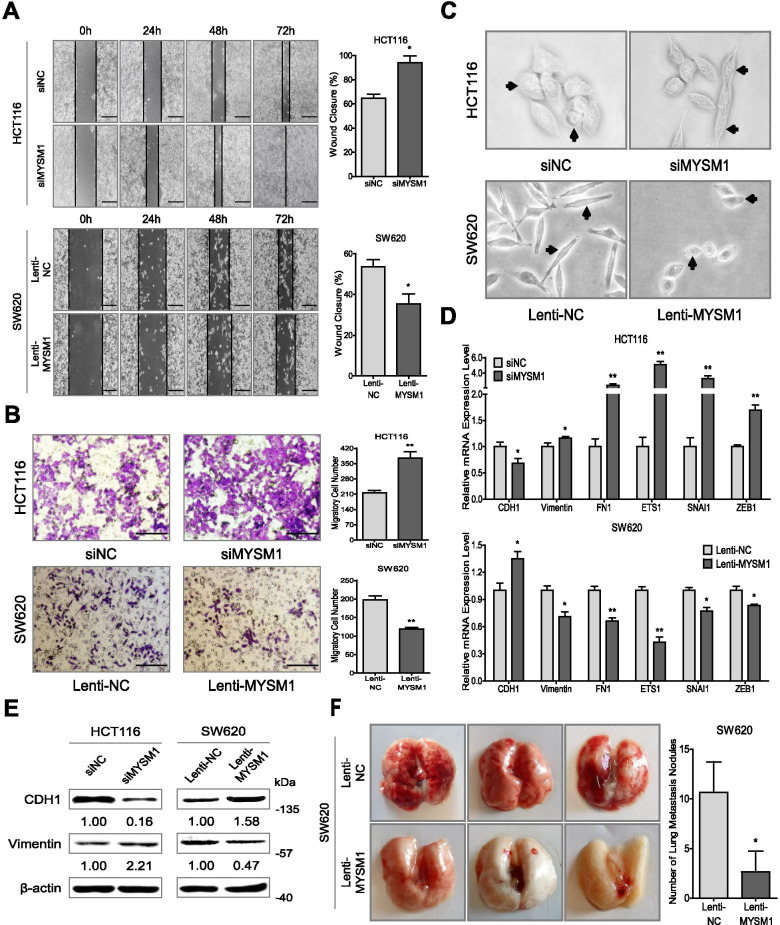
MYSM1 inhibits CRC cell growth in vitro. **A** Analysis of MYSM1 knockdown or overexpression efficiency at the mRNA level (*upper*) and protein level (*lower*). The data are shown as the means ± SDs (***P < 0.001, *n* = 3 independent experiments). **B** Cells with MYSM1 knockdown or overexpression were analyzed with a growth curve assay. The error bars indicate the SDs (*P < 0.05 and **P < 0.01, *n* = 6 independent experiments). **C** The expression and localization of MYSM1 (*red*) or PCNA (*red*) were determined by IF assay. The nuclei were counterstained with DAPI, and the significant differences in MYSM1 or PCNA staining are marked by *white arrows* (*n* = 3 independent experiments). *Scale bars:* 10 μm. **D** Western blot analysis of PCNA expression in MYSM1-knockdown HCT116 cells or MYSM1-overexpressing SW620 cells. **E** and **F** Cell cycle progression (**E**) and apoptosis (**F**) were analyzed in HCT116 cells transfected with siMYSM1/siNC and SW620 cells infected with Lenti-MYSM1/Lenti-NC by flow cytometry. The data are shown as the means ± SDs (*P < 0.05 and **P < 0.01, *n* = 3 independent experiments)

Then, murine CRC CT26 cells with Mysm1 knocked out via CRISPR-Cas9 (Additional file 12: Figure S2A and B) and SW620 cells infected with a lentivirus to construct stable MYSM1-overexpressing cell lines (Fig. 3A) were subcutaneously injected into athymic BALB/c mice. The growth of the implanted tumors was consistently measured (every 2–3 days), and the volume of the neoplasms was measured following neoplasm removal after 3 or 4 weeks (Additional file 12: Figure S2C and D). The results showed that neoplasm growth was greatly enhanced by Mysm1 knockout but markedly suppressed by MYSM1 overexpression in vivo (Fig. 1D). Taken together, these data confirm that MYSM1 is negatively correlated with CRC development and indicate that it may serve as a tumor suppressor in vivo.

To test whether MYSM1 is related to patient survival, we explored data from The Human Protein Atlas (https://www.proteinatlas.org) for a series of tumors. First, we divided the patients into a high-MYSM1-expression group and a low-MYSM1-expression group (Additional file 13: Figure S3A). The 5-year survival rate in the high-MYSM1-expression group was significantly higher than that in the low-MYSM1-expression group, especially among the CRC, COAD and READ patients (Additional file 13: Figure S3B). Similarly, using Gene Expression Omnibus (GEO, https://www.ncbi.nlm.nih.gov/geo/) data, we found that the patients in the high-MYSM1-expression group displayed better overall survival (OS) than those in the low-MYSM1-expression group (Fig. 1E and Additional file 13: Figure S3C), even patients with different stages of disease (Additional file 13: Figure S3D). These results suggest that a low MYSM1 level is closely correlated with a poor prognosis in several types of cancer, particularly CRC.

### MYSM1 restrains proliferation and promotes apoptosis in CRC cells in vitro

To further verify the antitumor role of MYSM1 in CRC, we conducted a series of in vitro gain- and loss-of-function assays in the SW620 and HCT116 cell lines, which have relatively low and relatively high MYSM1 expression, respectively (Additional file 11: Figure S1C). The expression of MYSM1 in these cell lines was either suppressed by transient transfection with a pool of specific small interfering RNAs (siRNAs) or enhanced via infection with Lenti-MYSM1 particles (Fig. 3A). A growth curve assay indicated that among HCT116 cells, those transfected with MYSM1 siRNA (siMYSM1) for MYSM1 downregulation exhibited greater proliferation than those transfected with NC siRNA (siNC). In contrast, among SW620 cells, the cells infected with the MYSM1 lentivirus (Lenti-MYSM1) for MYSM1 overexpression exhibited less proliferation than the cells infected with the NC lentivirus (Lenti-NC) (Fig. 3B). Consistent with this result, PCNA expression was dramatically reduced in Lenti-MYSM1-infected SW620 cells (Fig. 3C and D) and increased in siMYSM1-transfected HCT116 cells (Fig. 3D). Notably, the IF staining results showed that MYSM1 was expressed mainly in the nucleus, which was consistent with our IHC results in clinical samples and previous literature [3, 25], indicating that MYSM1 functions mainly in the nucleus.

HCT116 cells with transient knockdown of MYSM1 and SW620 cells with stable MYSM1 overexpression were then synchronized at the G_0_/G_1_ phase of the cell cycle by serum-free starvation for 24 h followed by cultivation in serum-supplemented medium for an additional 48 h. Compared with siNC treatment, siMYSM1 treatment in HCT116 cells was associated with a remarkable increase in the proportion of S-phase cells along with a decrease in the proportion of G_0_/G_1_-phase cells; however, compared with Lenti-NC treatment, Lenti-MYSM1 treatment in SW620 cells resulted in a notable reduction in the proportion of S-phase cells and an increase in the proportion of G_0_/G_1_-phase cells (Fig. 3E). In addition, flow cytometry analysis with propidium iodide (PI) and Annexin V-FITC staining showed that the downregulation of MYSM1 dramatically prevented apoptosis in HCT116 cells, whereas the overexpression of MYSM1 promoted apoptosis in SW620 cells (Fig. 3F). We observed similar and consistent results in cell proliferation, cell cycle and apoptosis assays on CT26 cells with genetic MYSM1 knockout, SW480 cells with transient MYSM1 knockdown and LOVO cells with MYSM1 overexpression (Additional file 12: Figure S2E and Additional file 14: Figure S4A-C). Altogether, the results of these experiments suggest that MYSM1 is able to inhibit cell proliferation and induce apoptosis in CRC cells in vitro.

### MYSM1 suppresses the migration of CRC cells and prevents the epithelial-mesenchymal transition (EMT) process

**Fig. 4:**
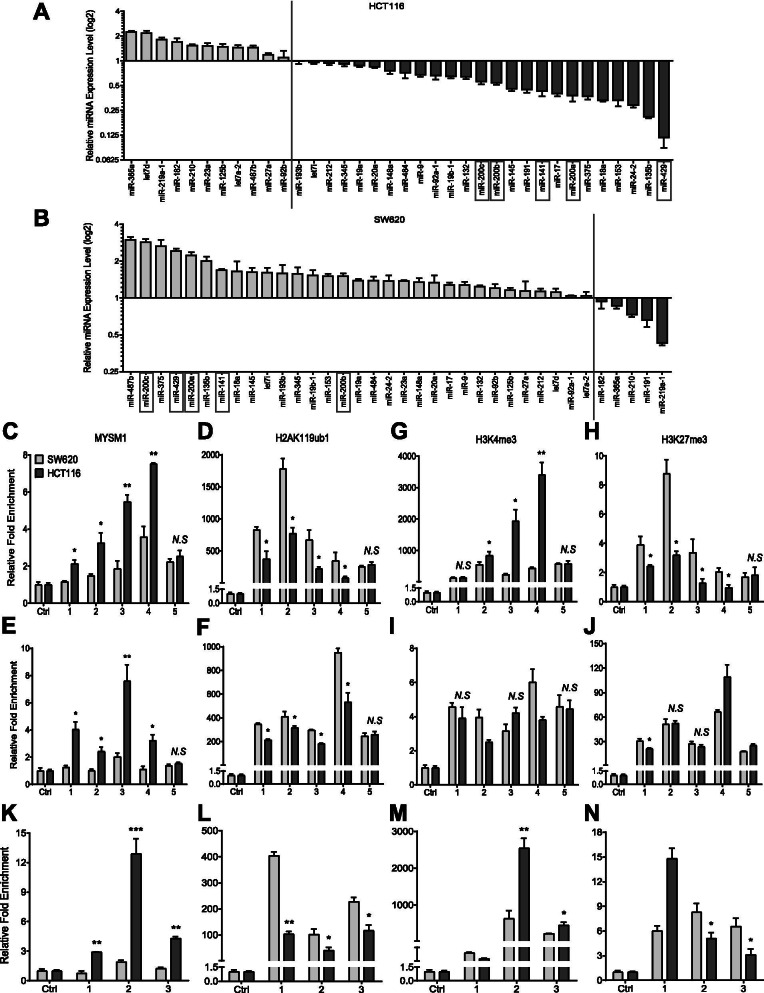
MYSM1 suppresses CRC cell migration and the EMT process. **A** and **B** The metastatic ability of HCT116 cells transfected with siMYSM1/siNC and SW620 cells infected with Lenti-MYSM1/Lenti-NC was tested in wound healing (**A**) and Transwell (**B**) assays. *Scale bars:* 50 μm. The error bars indicate the SDs (*P < 0.05 and **P < 0.01, *n* = 3 independent experiments). **C** Morphological observations of cells with MYSM1 knockdown or overexpression under a light microscope. **D** Expression analysis of six representative EMT biomarkers in HCT116 cells transfected with siMYSM1/siNC and SW620 cells infected with Lenti-MYSM1/Lenti-NC by qRT-PCR. The data are shown as the means ± SDs (*P < 0.05 and **P < 0.01, *n* = 3 independent experiments). **E** The protein levels of CDH1 and vimentin in HCT116 cells transfected with siMYSM1/siNC and SW620 cells infected with Lenti-MYSM1/Lenti-NC were analyzed by western blot. **F** Engineered SW620 cells were injected into the tail veins of athymic BALB/c mice to investigate the metastatic potential of CRC cells. After 6 weeks, the lungs were photographed (*left*), and the nodules were counted (*right*). The data are expressed as the means ± SDs (*P < 0.05, *n* = 3 independent experiments)

Based on the role of EMT in CRC development and progression, we investigated the potential role of MYSM1 in EMT prevention. Knockdown of MYSM1 significantly elevated migration in HCT116 cells, whereas overexpression of MYSM1 dramatically decreased cell migration in SW620 cells, as measured by wound healing (Fig. 4A) and Transwell assays (Fig. 4B). The engineered CT26, SW480 and LOVO cells displayed results similar to those observed in HCT116 and SW620 cells (Additional file 14: Figure S4D and E). Moreover, downregulation of MYSM1 induced a morphological transformation of epithelioid HCT116 cells into spindle-shaped and fibroblast-like cells with looser cell connections; in contrast, overexpression of MYSM1 caused SW620 cells to grow more closely together and clearly adopt an epithelial phenotype (Fig. 4C).

To further identify whether MYSM1 plays a role in EMT, the effects of increases or decreases in MYSM1 expression on EMT biomarkers were tested in HCT116 or SW620 cells, respectively. The qRT-PCR data revealed that the downregulation of MYSM1 in HCT116 cells resulted in significant increases in EMT biomarkers, such as vimentin, FN1, ETS1, SNAI1 and ZEB1, along with a decrease in CDH1 at the mRNA level. In contrast, we observed opposite expression patterns of these six EMT biomarkers when MYSM1 was upregulated in SW620 cells (Fig. 4D). Furthermore, reducing the expression of MYSM1 suppressed the protein levels of the epithelial biomarker CDH1 and concomitantly increased the levels of the mesenchymal biomarker vimentin in HCT116 cells. The opposite effects were observed in SW620 cells (Fig. 4E). Importantly, in a mouse model of tumor metastasis, there were smaller and fewer metastatic nodules in the lungs of mice treated with Lenti-MYSM1-infected SW620 cells than in those of mice treated with Lenti-NC-infected SW620 cells (Fig. 4F). Furthermore, the livers of the mice in the Lenti-NC treatment group showed more serious lesions than those of the mice in the Lenti-MYSM1 group (Additional file 14: Figure S4F). In conclusion, these findings suggest that MYSM1 participates in preventing the EMT process and impairing CRC cell migration.

### MYSM1 epigenetically facilitates the expression of miR-200 family members and CDH1

**Fig. 5:**
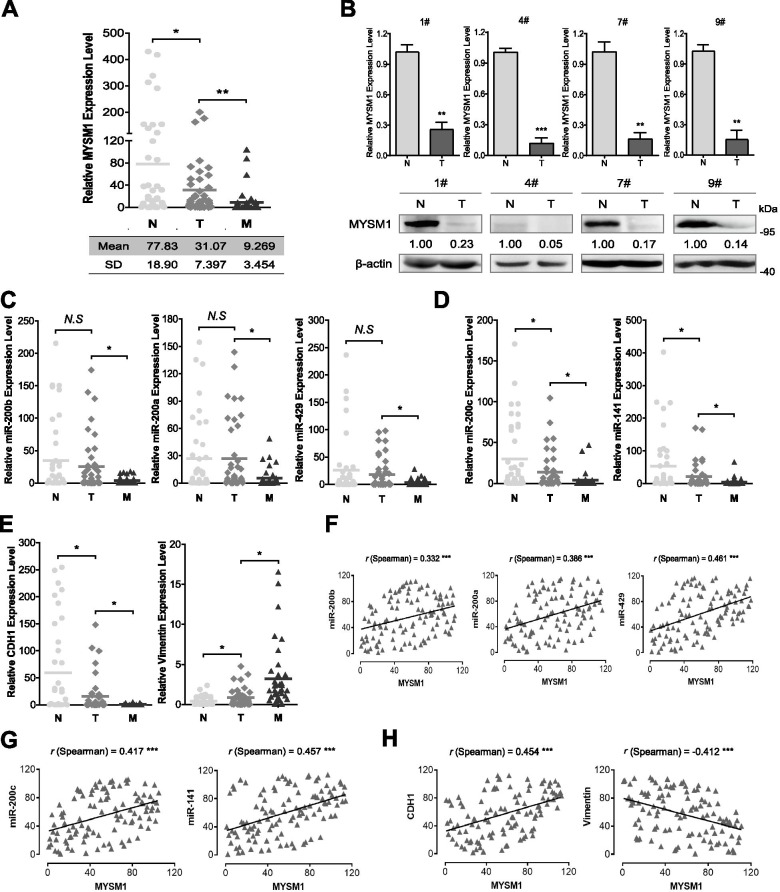
MYSM1 directly binds to the promoter regions of miR-200 family members and CDH1 to initiate transcription. **A** and **B** qRT-PCR analysis of the alterations in the expression levels of 36 miRNAs following MYSM1 knockdown in HCT116 cells (**A**) or overexpression in SW620 cells (**B**). The data were logarithmically transformed (log2) and are shown as the means ± SDs (*n* = 3 independent experiments). The dotted line divides the data (< 1 or > 1). **C-N** ChIP analysis of the enrichment in the promoter regions of miR-200b-a-429, miR-200c-141 and CDH1 using IgG (Ctrl) and specific MYSM1 (**C**, **E** and **K**), H2AK119ub1 (**D**, **F** and **L**), H3K4me3 (**G**, **I** and **M**) and H3K27me3 (**H**, **J** and **N**) antibodies in untreated HCT116 and SW620 cells. The data are shown as the means ± SDs (*N. S.*, no significance; *P < 0.05 and **P < 0.01, *n* = 3 independent experiments)

miRNAs are vital regulators of gene expression, and many aberrantly expressed miRNAs are associated with cancer progression. To investigate whether miRNAs are involved in MYSM1-mediated CRC suppression, we analyzed the expression of a panel of 36 selected miRNAs that have been reported to be closely related to CRC. Because MYSM1 can relieve transcriptional repression by H2AK119ub1, we focused on miRNAs that were consistent with MYSM1 expression changes. We noticed that when MYSM1 was knocked down in HCT116 cells, 19 miRNAs were significantly downregulated; in contrast, 25 miRNAs were notably upregulated when MYSM1 was overexpressed in SW620 cells (Additional file 15: Figure S5A and B). Intriguingly, among the altered miRNAs, the miR-200 family members, namely, miR-200b, miR-200a, miR-429, miR-200c and miR-141, were significantly changed (Fig. 5A and B).

The miR-200 family members, which are located in clusters on chromosome 1 (miR-200b, miR-200a and miR-429) and chromosome 12 (miR-200c and miR-141), are implicated in cell survival suppression and EMT in CRC [36, 37]. To further explore the regulatory mechanisms connecting MYSM1 and the miR-200 family members, we performed ChIP assays with five independent primers to specifically amplify the promoter regions of miR-200b-a-429 and miR-200c-141 (Additional file 16: Figure S6A). The results showed that compared with SW620 cells, HCT116 cells, which contain relatively higher levels of endogenous MYSM1, had more MYSM1 enrichment (Fig. 5C and E) and less H2AK119ub1 occupancy (Fig. 5D and F) in the promoter regions of miR-200b-a-429 and miR-200c-141. Furthermore, the promoter region of miR-200b-a-429 was markedly occupied by H3K4me3 in HCT116 cells (Fig. 5G), whereas in SW620 cells, more H3K27me3 enrichment was observed (Fig. 5H). However, there were no significant differences in these two epigenetic markers in the promoter region of miR-200c-141 between HCT116 and SW620 cells (Fig. 5I and J).

Notably, we also found that MYSM1 was able to bind to the promoter region of CDH1 (Fig. 5K) and remove H2AK119ub1 in order to relieve transcriptional repression (Fig. 5L). More enrichment of H3K4me3 and less occupancy of H3K27me3 were observed in the CDH1 promoter region in the HCT116 cells than in the SW620 cells (Fig. 5M and N), without major impacts on the entire transcriptome (Additional file 16: Figure S6B). These data suggest that MYSM1 directly binds the promoter regions of miR-200 family members and CDH1 and impair the enrichment of H2AK119ub1 in order to promote miR-200 family/CDH1 transcription.

### miR-200 family members and CDH1 are positively correlated with MYSM1 in CRC cells and patient specimens in vivo

To further clarify the relationship between the expression level of MYSM1 and the expression levels of miR-200 family members and CDH1, we first detected the expression of these molecules in CRC cell lines. The levels of miR-200 family members and CDH1 were notably higher in epithelial HCT116 or SW480 cells than in mesenchymal SW620 cells (Additional file 15: Figure S5C and D) and were positively correlated with endogenous MYSM1 levels (Additional file 11: Figure S1C). In contrast, vimentin was markedly expressed in SW620 cells (Additional file 15: Figure S5D).

**Fig. 6:**
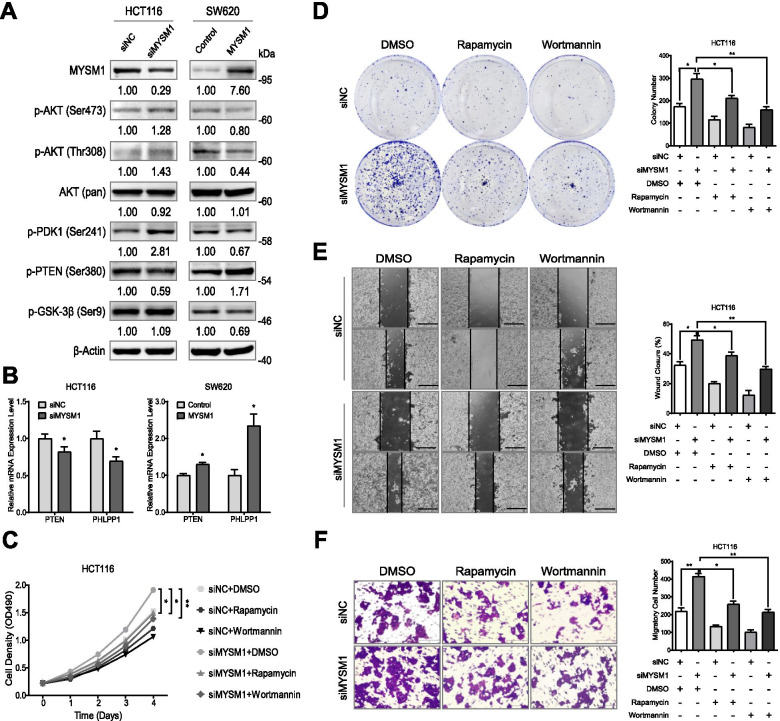
MYSM1 is positively correlated with miR-200 family members and CDH1 in vivo. **A** MYSM1 is differentially expressed in paired adjacent normal (N), primary tumor (T) and metastatic (M) tissues from 41 CRC patients. Scatter plot showing the relative MYSM1 mRNA level. The statistical data (means and SDs) are shown in the table (*right*). **B** Representative data of MYSM1 expression patterns at the mRNA level (*upper*) and protein level (*lower*) in CRC patients. **C-E** Scatter plots of the miR-200b, miR-200a and miR-429 levels (**C**); the miR-200c and miR-141 levels (**D**); and the CDH1 and vimentin mRNA levels (**E**) in 41 paired CRC patient specimens. In **A-E**, the data are shown as the means ± SDs (*N. S.*, no significance; *P < 0.05, **P < 0.01 and ***P < 0.001, *n* = 3 independent experiments). **F-H** Correlation analyses of MYSM1 with the miR-200b cluster (**F**), MYSM1 with the miR-200c cluster (**G**) and MYSM1 with CDH1 (*left*) and vimentin (*right*) (**H**). The Spearman rank correlation test was used to determine the statistical significance. The data are presented as the correlation coefficient “*r*” (***P < 0.001)

We next determined the expression levels of MYSM1 in 41 CRC patient samples, including paired samples of the adjacent normal mucosa (N), primary tumor tissue (T) and metastatic lymph node or hepatic tumor (M) tissue. The results revealed that the relative MYSM1 expression level in normal mucosa (77.83 ± 18.90) was dramatically higher than that in primary tumors (31.07 ± 7.397) or metastatic lymph nodes (9.269 ± 3.454) at the mRNA level (Fig. 6A) and protein level (Fig. 6B), which was consistent with the findings of the database analysis and in vitro experiments, indicating an intrinsic tumor suppressor role of MYSM1 in CRC in vivo.

To further elucidate the positive association between MYSM1 and miR-200 family members/CDH1 in vivo, we detected the relative expression levels of miR-200b, miR-200a, miR-429 (Fig. 6C), miR-200c, miR-141 (Fig. 6D), CDH1, and vimentin (Fig. 6E) in 41 paired patient specimens. Our results showed that the expression of the miR-200 family members and CDH1 was substantially repressed in metastatic lymph nodes compared with normal mucosa and primary tumors, but vimentin exhibited the opposite expression trend (Fig. 6C-E). In addition, the levels of miR-200c, miR-141 and CDH1 in primary tumors were relatively lower than those in normal mucosa, but the levels of miR-200b, miR-200a and miR-429 did not significantly differ between the primary tumors and normal tissues. Furthermore, Spearman rank correlation analysis revealed that MYSM1 expression was positively correlated with miR-200b (*r* = 0.332), miR-200a (*r* = 0.386), miR-429 (*r* = 0.461) (Fig. 6F), miR-200c (*r* = 0.417), miR-141 (*r* = 0.457) (Fig. 6G) and CDH1 (*r* = 0.454) expression but negatively correlated with vimentin (*r* = − 0.412) expression (Fig. 6H). Taken together, these results strongly indicate that a positive relationship exists between MYSM1 and miR-200 family members and between MYSM1 and CDH1, thus providing evidence of the antitumor role of MYSM1.

### miR-200 family members participate in MYSM1-mediated CRC suppression

Since miR-200 family members have been reported to suppress CRC progression, we investigated whether miR-200 family members play a role in the antitumor effect of MYSM1. For this purpose, miR-200b or miR-200c was overexpressed in HCT116 cells or knocked down in SW620 cells by transient transfection (Additional file 16: Figure S6C and D), and the impact of MYSM1 gain- or loss-of-function on the tumorigenic potential of these cells was measured in a series of assays. The increased proliferation potential induced by silencing MYSM1 was partially diminished when miR-200b or miR-200c was concomitantly overexpressed in HCT116 cells (Additional file 17: Figure S7A), and the reduced growth capacity observed following MYSM1 enhancement was eliminated when miR-200b or miR-200c was simultaneously knocked down in SW620 cells (Additional file 17: Figure S7B) in the growth curve assay. In addition, interfering with MYSM1 expression promoted colony formation, but this effect was reversed by miR-200b or miR-200c mimic transfection in HCT116 cells (Additional file 17: Figure S7C), whereas cotransfection with inhibitors of miR-200b or miR-200c reversed the pMSCV-MYSM1 vector-mediated loss of colony formation ability in SW620 cells (Additional file 17: Figure S7D). Although the disruption of MYSM1 expression markedly enhanced HCT116 cell migration, exogenous expression of miR-200b or miR-200c partially abolished this stimulatory effect (Additional file 17: Figure S7E). Similarly, the elevation in MYSM1 significantly impaired the metastatic capabilities of SW620 cells, whereas repression of endogenous miR-200b or miR-200c effectively reversed the MYSM1-induced suppression of migration in the Transwell assays (Additional file 17: Figure S7F). Collectively, these data indicate that MYSM1 inhibits tumor progression in CRC cells by activating miR-200 family members.

### The suppressive effect of MYSM1 contributes to functional loss of PI3K/AKT signaling and reduced activation of EMT

**Fig. 7:**
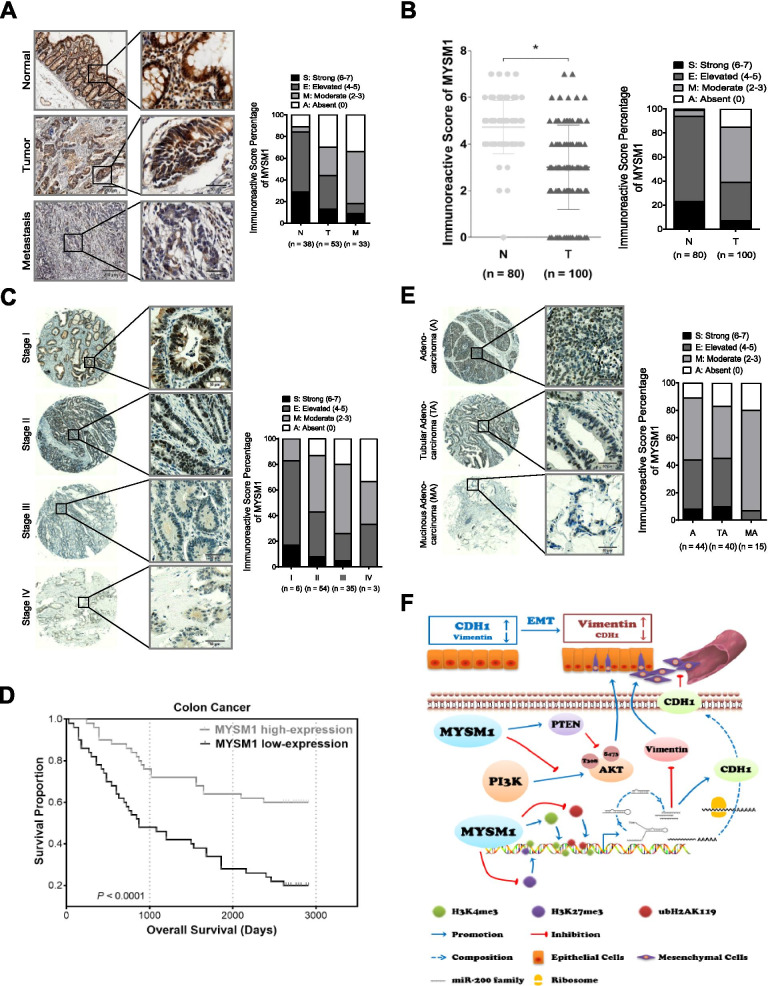
MYSM1 suppresses CRC tumorigenesis by inhibiting PI3K/AKT signaling. **A** Western blot analysis showing that PI3K/AKT signaling, as indicated by the expression of p-AKT (Ser473), p-AKT (Thr308), p-PDK1 (Ser241) and p-GSK-3β (Ser9), was stimulated in HCT116 cells (*left*) with MYSM1 knockdown and inhibited in SW620 cells (*right*) with MYSM1 overexpression, while PTEN presented the opposite trends. **B** The mRNA levels of PTEN and PHLPP1 in pretreated HCT116 and SW620 cells were analyzed by qRT-PCR. **C** and **D** The proliferation of HCT116 cells pretreated with siNC or siMYSM1 accompanied by DMSO, rapamycin (10 nmol/L) or wortmannin (100 nmol/L) as indicated for 48 h was examined by growth curve (**C**) and colony formation (**D**) assays. **E** and **F** Migration in pretreated HCT116 cells was determined via wound healing (**E**) and Transwell (**F**) assays. *Scale bars:* 50 μm. In **B-F**, the data are shown as the means ± SDs (*P < 0.05 and **P < 0.01, *n* = 3 independent experiments)

The MYSM1-mediated inhibition of NF-κB through the inactivation of TRAF3 and TRAF6 complexes in innate immunity [31] led us to speculate that MYSM1 is likely involved in the regulation of PI3K/AKT signaling since NF-κB is among the functional downstream targets in this pathway. MYSM1 gain- and loss-of-function experiments showed that when MYSM1 was knocked down in HCT116 cells, proteins representing active PI3K/AKT signaling, such as p-AKT (Ser473), p-AKT (Thr308), p-PDK1 (Ser241) and p-GSK-3β (Ser9), were significantly enhanced; however, the expression of PTEN, an inhibitor of PI3K/AKT signaling, was lower in the knockdown group than in the control group. In contrast, overexpression of MYSM1 in SW620 cells led to the opposite results, as the active markers were dramatically suppressed, while PTEN exhibited a stronger signal (Fig. 7A). Furthermore, the mRNA levels of PTEN and PHLPP1, two negative regulators of PI3K/AKT signaling, paralleled the changes in MYSM1 expression (Fig. 7B). These observations support the argument that MYSM1 participates in and suppresses PI3K/AKT signaling.

We next introduced the mTOR1 inhibitor rapamycin (10 nmol/L) and the PI3K suppressor wortmannin (100 nmol/L) to further verify the functional loss incurred by MYSM1 on PI3K/AKT signaling. Indeed, the results showed that deletion of MYSM1 expression activated the PI3K/AKT signaling pathway, but the changes in the protein levels of p-AKT (Ser473)/p-AKT (Thr308) were partially reversed by rapamycin or wortmannin 48 h post transfection (Additional file 16: Figure S6E). Stimulation of PI3K/AKT signaling mediated by transfection of siMYSM1 greatly enhanced the proliferative and metastatic capacities of HCT116 cells. Nevertheless, the siMYSM1-mediated increase in HCT116 cell proliferation was partially reduced by rapamycin or wortmannin, as determined by growth curve (Fig. 7C) and colony formation (Fig. 7D) assays. Additionally, rapamycin and wortmannin reduced the PI3K/AKT signaling activation caused by MYSM1 downregulation and enhanced migration in HCT116 cells, as evidenced by wound healing (Fig. 7E) and Transwell (Fig. 7F) assays. In conclusion, these data suggest that MYSM1 inhibits PI3K/AKT signaling and thus reveal another mechanism by which MYSM1 represses tumorigenesis in CRC cells.

### Prognostic and diagnostic value of MYSM1 in CRC

We previously used multiple online databases to analyze the relationship between MYSM1 expression and CRC patient survival. To further elucidate the prognostic value of MYSM1 in CRC, we performed IHC of tissue microarrays with more clinical samples to illuminate the antimetastatic function of MYSM1. A tissue microarray containing 38 adjacent normal tissues (N), 53 adenocarcinomas (T) and 33 lymph node metastatic carcinomas (M) was analyzed and revealed that MYSM1 expression, which was predominantly in the nucleus, was significantly higher in adjacent normal tissues and lower in lymph node metastatic carcinomas than in primary adenocarcinomas (Fig. 2A and Table 1). In addition, MYSM1 was negatively associated with the CRC stage (≤ IIIB or ≥ IIIC, *P* = 0.032) and the tumor stage (≤ T_3_ or T_4_, *P* = 0.026) (Table 1). In another tissue microarray of 100 carcinomas (T), including 80 samples harboring paired adjacent normal tissues (N), we detected the survival potential represented by MYSM1 expression. Consistent with the previous observations, the immunoreactivity scores of MYSM1 in the carcinomas were found to be significantly lower than those in the adjacent normal tissues (Fig. 2B), and MYSM1 expression was inversely related to the CRC stage (Fig. 2C and Table 2). In addition, MYSM1 downregulation was observed in kidney and brain tumors (Additional file 18: Figure S8A-C).

**Table 1:** Correlation between MYSM1 expression and clinicopathological features of CRC patients in the tissue microarray of metastasis

	Total		Low MYSM1		High MYSM1			
Variable	N	%	N	%	N	%	*χ*^*2*^	***P*** Value^a^
Pathological Diagnosis								
Adjacent Normal Tissue	38	30.7	6	9.5	32	52.5	32.054	**< 0.001**
Adenocarcinoma	53	42.7	30	47.6	23	37.7		
Lymph Node Metastasis	33	26.6	27	42.9	6	9.8		
Adjacent Normal Tissue	38	41.8	6	16.7	32	58.2	15.419	**< 0.001**
Adenocarcinoma	53	58.2	30	83.3	23	41.8		
Adenocarcinoma	53	61.6	30	52.6	23	79.3	5.785	**0.016**
Lymph Node Metastasis	33	38.4	27	47.4	6	20.7		
Adjacent Normal Tissue	38	53.5	6	18.2	32	84.2	30.955	**< 0.001**
Lymph Node Metastasis	33	46.5	27	81.8	6	15.8		
Location								
Colon	45	84.9	28	93.3	17	73.9	3.831	0.050
Rectum	8	15.1	2	6.7	6	26.1		
Age (y)								
< 40	8	15.1	5	16.7	3	13.0	1.065	0.587
40–60	28	52.8	14	46.7	14	60.9		
> 60	17	32.1	11	36.6	6	26.1		
Gender								
M	38	71.7	21	70.0	17	73.9	0.098	0.754
F	15	28.3	9	30.0	6	26.1		
Grade								
I	10	18.9	8	26.7	2	8.7	2.783	0.249
II	26	49.1	13	43.3	13	56.5		
III	17	32.0	9	30.0	8	34.8		
Stage								
≤ IIIB	44	83.0	22	73.3	22	95.7	4.600	**0.032**
≥ IIIC	9	17.0	8	26.7	1	4.3		
Tumor Stage								
≤ T_3_	35	66.0	16	53.3	19	82.6	4.975	**0.026**
T_4_	18	34.0	14	46.7	4	17.4		
Nodal Stage								
N_0_	13	24.5	9	30.0	4	17.4	1.759	0.415
N_1_	27	51.0	13	43.3	14	60.9		
N_2_	13	24.5	8	26.7	5	21.7		

^a^The *P* values were calculated in SPSS 25.0 using a chi-square test

*P* values < 0.05 were considered to indicate statistical significance

**Table 2:** Correlation between MYSM1 expression and clinicopathological features of CRC patients in the tissue microarray of survival

	Total		Low MYSM1		High MYSM1			
Variable	N	%	N	%	N	%	*χ*^*2*^	***P*** Value^a^
Survival								
Alive	40	40.0	11	18.0	29	74.4	31.449	**< 0.001**
Deceased	60	60.0	50	82.0	10	25.6		
Pathological Diagnosis								
Adjacent Normal Tissue	80	44.4	5	7.6	75	65.8	57.370	**< 0.001**
Carcinoma	100	55.6	61	92.4	39	34.2		
Histologic Subtypes								
Adenocarcinoma	44	44.4	25	41.0	19	50.0	7.549	**0.023**
Tubular Adenocarcinoma	40	40.4	22	36.1	18	47.4		
Mucinous Adenocarcinoma	15	15.2	14	22.9	1	2.6		
Location								
Ascending Colon	33	33.7	23	38.3	10	26.3	5.706	0.127
Transverse Colon	26	26.5	18	30.0	8	21.1		
Descending Colon	11	11.2	7	11.7	4	10.5		
Rectosigmoid Colon	28	28.6	12	20.0	16	42.1		
Age (y)								
≤ 60	21	22.3	11	18.6	10	28.6	2.040	0.361
60–80	57	60.7	39	66.1	18	51.4		
≥ 80	16	17.0	9	15.3	7	20.0		
Gender								
M	54	54.5	35	58.3	19	48.7	0.881	0.348
F	45	45.5	25	41.7	20	51.3		
Grade								
< II	7	7.0	4	6.5	3	7.7	2.679	0.262
< III	78	78.0	45	73.8	33	84.6		
≥ III	15	15.0	12	19.7	3	7.7		
Stage								
1	6	6.1	1	1.7	5	13.2	7.902	**0.048**
2	54	55.1	31	51.7	23	60.5		
3	35	35.7	26	43.3	9	23.7		
4	3	3.1	2	3.3	1	2.6		
Tumor Stage								
≤ T_2_	7	7.3	2	3.4	5	13.5	6.741	**0.034**
T_3_	75	78.1	45	76.3	30	81.1		
T_4_	14	14.6	12	20.3	2	5.4		
Nodal Stage								
N_0_	60	61.2	32	53.3	28	73.7	5.994	0.050
N_1_	27	27.6	18	30.0	9	23.7		
N_2_	11	11.2	10	16.7	1	2.6		
Metastasis								
M_0_	97	97.0	59	96.7	38	97.4	0.042	0.838
M_1_	3	3.0	2	3.3	1	2.6		

^a^The *P* values were calculated in SPSS 25.0 using a chi-square test

*P* values < 0.05 were considered to indicate statistical significance

We then divided 100 CRC samples into a high-MYSM1-expression group (*n* = 50) and a low-MYSM1-expression group (*n* = 50) according to the MYSM1 immunoreactive score. Kaplan-Meier analysis showed that the OS of patients with high MYSM1 expression in CRC was significantly better than that of patients with low MYSM1 expression (Fig. 2D and Table 2). Moreover, upon analyzing the different histological subtypes, we found that compared with tubular adenocarcinoma (TA) or adenocarcinoma (A), mucinous adenocarcinoma (MA), which is among the worst pathological subtypes in terms of the clinical prognosis of CRC, barely expressed MYSM1 (Fig. 2E and Table 2). Similarly, MYSM1 was inversely correlated with the CRC stage and tumor stage, as shown in both Tables 1 and 2. Importantly, ROC curve analysis on 41 paired specimens revealed that although MYSM1 did not differentiate between primary CRC tumors and normal control tissue, with an AUC of 0.570 (*P* = 0.277), the possibility of metastasis was identifiable, with an AUC of 0.735 (*P* <  0.001) (Additional file 18: Figure S8D). These findings indicate that the MYSM1 expression level is a useful index for the diagnosis of CRC metastasis.

Collectively, our data presented here strongly support the role of MYSM1 in inhibiting tumorigenesis and suggest that MYSM1 may serve as a biomarker for CRC diagnosis and a promising target for cancer therapy.

## Discussion

Although the association between MYSM1 and cancer remains obscure, some clues indicate that MYSM1 is involved in tumorigenesis. First, MYSM1 suppresses the cell cycle and proliferation. Wang T reported that MYSM1 deficiency drives HSCs from quiescence into rapid cycling [26]. Similarly, a recent study showed that MYSM1 can suppress proliferation in B1a cells through a miRNA-associated axis [38]. In our present study, we found that MYSM1 dramatically suppresses cell cycle progression and proliferation while stimulating apoptosis in CRC cells or xenografts. Second, in a previous study, an increased incidence of lymphoma was observed in Mysm1 knockout mice [39]. We and other groups also revealed an antitumor effect of MYSM1 in prostate cancer and renal carcinoma [40, 41]. These results prompted the hypothesis that the loss of MYSM1 might predispose human patients to cancer. In addition, BAP1 and USP16, both of which are H2AK119ub1 DUBs, have antitumor functions and are often absent or mutated in multiple solid tumors and myeloid malignancies [6, 20, 22, 42]. MYSM1, which possesses zinc metalloprotease activity to remove ubiquitin on H2AK119, likely performs functions similar to those of BAP1 and USP16. In summary, the above clues strongly suggest that MYSM1 plays a role in CRC.

Our findings in the present study provide additional clues that enhance our understanding of the antioncogenic potential of MYSM1. In this regard, the in vitro and in vivo studies indicate that the expression level of MYSM1 in primary tumor tissues is lower than that in adjacent normal tissues and is positively correlated with the OS of CRC patients, demonstrating the negative regulatory interaction between MYSM1 and CRC. However, these results are in contrast to Li Y’s report. Li Y and his colleagues reported that MYSM1 expression is significantly elevated in carcinoma tissues and is associated with tumor progression in CRC [43]. We speculate that the main reason for these contradictory results is the different antibodies used for IHC in these two studies. The intracellular localization of MYSM1 in either the nucleus or cytoplasm is strictly regulated. Nuclear MYSM1 constitutively functions as a housekeeping gene in H2A deubiquitination [3], whereas cytoplasmic MYSM1 displays different features and is temporally regulated during inflammation [31]. Notably, MYSM1 was detected mainly in the nucleus with an IHC antibody from Sigma-Aldrich (#HPA054291) in our study; however, in Li Y’s study, a different antibody from Abcam (#180570) was used, and MYSM1 was predominantly detected in the cytoplasm, especially in the IHC sections of intestinal villi and glands. We performed further IHC and confirmed that the subcellular localization of MYSM1 was different with these different antibodies, indicating that nonspecific staining might have led to the conflicting conclusions (Additional file 18: Figure S8E).

A potential correlation exists between MYSM1 and PI3K/AKT signaling, as supported by some studies. Among the posttranslational modifications, ubiquitination is a key promoter of AKT activation [44]. Upon stimulation with growth factors, E3 ligases can ubiquitinate the PH domain of AKT, which mediates AKT translocation to the plasma membrane for further activation of downstream biological functions, e.g., glycolysis and tumorigenesis [45, 46]. Interaction between PI(3,4,5)P3 and the PH domain of AKT is also a general mechanism for recruitment of AKT to the cellular membrane and AKT phosphorylation, which is induced by TRAF6; these processes prompt AKT ubiquitination at K8 and K14 within the PH domain [44]. Nonetheless, as a critical member of the DUB family, MYSM1 can bind and inactivate TRAF3 and TRAF6 complexes through its SWIRM and MPN domains, thus suppressing the NF-κB-mediated overreaction of innate immunity [31]. Additionally, NF-κB is an important downstream transcription factor for PI3K/AKT signaling, which helps convey and expand the activating effects of AKT phosphorylation. Moreover, MYSM1 can remove the polyubiquitination chain at the K63, K27 and M1 loci in PI(4,5)P2 triggered by NOD2 signaling, thus inhibiting peritonitis, systemic inflammatory responses and liver injury [30]; this indicates that MYSM1 can probably regulate AKT at an upstream site, which further suggests that MYSM1 is involved in the transduction of PI3K/AKT signaling.

Previous studies have reported that MYSM1 functions mainly by deubiquitinating histone H2A. In the present work, we found that MYSM1 may directly bind to the promoter regions of miR-200 family members and CDH1 to initiate the transcription of these molecules while simultaneously inhibiting the PI3K/AKT signal transduction pathway. The miR-200 family plays important regulatory roles in preventing tumorigenesis, EMT and metastasis in CRC [36, 47, 48]. In contrast, PI3K/AKT signaling, which participates in diverse cellular events, including by promoting the cell cycle, proliferation, and migration and inhibiting apoptosis, markedly stimulates cancer progression [49]. Intriguingly, miR-200 family members may either prevent PI3K/AKT signal transduction [50] or be suppressed by the PI3K/AKT signaling pathway [51], thus forming a negative feedback loop. In our study, we demonstrated the direct suppressive effects of MYSM1 deletion on the miR-200 family and CDH1. Although the inhibitory influence of MYSM1 on PI3K/AKT signaling has also been confirmed, the underlying mechanisms, including those related and unrelated to the miR-200 family, still need to be further explored.

## Conclusions

In summary, our data reveal that MYSM1 plays a suppressor role in CRC and that its strong expression is associated with favorable prognosis. We found that MYSM1 might epigenetically enhance the expression of the miR-200 family and CDH1 and inhibit PI3K/AKT signaling (Fig. 2F) to suppress CRC oncogenesis and progression. Furthermore, we also demonstrate that MYSM1 expression is significant for the clinical assessment of prognosis and that it may be a promising prognostic biomarker and new therapeutic target for CRC.

## Supplementary Information

**Additional file 1: Table S1.** Information of CRC patients collected for clinical studies.

**Additional file 2: Table S2.** Information of CRC patients collected in tissue microarray of metastasis.

**Additional file 3: Table S3.** Information of CRC patients collected in tissue microarray of survival.

**Additional file 4: Table S4.** Oligonucleotide sequences in this study.

**Additional file 5: Table S5.** Antibodies used for ChIP, western blot, IHC and IF assays in this study.

**Additional file 6: Table S6.** Primer sequences for the 36 miRNAs in qRT-PCR assay of CRC cells.

**Additional file 7: Table S7.** Primer sequences for the miR-200 family members/CDH1 in ChIP assay.

**Additional file 8: Table S8.** Primers used for qRT-PCR analysis in this study.

**Additional file 9: Table S9.** Primers used for construction of plasmids in CRISPR-Cas9 assay.

**Additional file 10.** Supplementary methods.

**Additional file 11: Figure S1.** MYSM1 widely exists and is differentially expressed in normal vs. tumor tissues in the human body. **A** Representative IHC of MYSM1 in a series of normal tissues derived from different human organs. *Scale bars*: 200 μm. **B** Analysis of MYSM1 expression data from different tumor tissues and each corresponding adjacent normal tissue in the GEPIA database. **C** Analysis of MYSM1 mRNA levels in a variety of cell lines from different human tumors by qRT-PCR. The dark columns represent the cell lines with greater metastatic potential or a higher tumor grade. The dotted lines distinguish the cell lines on the basis of their different attributes (normal vs. tumor cells or cells with different migration potentials or grades). The data are shown as the means ± SDs (*n* = 3 independent experiments).

**Additional file 12: Figure S2.** MYSM1 suppresses CRC cell proliferation in vitro and in vivo. **A** Efficiency of Mysm1 knockout by CRISPR-Cas9 in CT26 cells. Mysm1 expression fragments in the genome were detected by PCR. **B** Analysis of MYSM1 mRNA and protein levels in differently treated cells (CT26 cells, CRISPR-Cas9; SW480 cells, transient knockdown; and LOVO cells, transient overexpression) via qRT-PCR (*upper*) and western blot (*lower*). The data are presented as the means ± SDs (**P <  0.01, *n* = 3 independent experiments). **C** Representative photographs of tumor-bearing mice that received subcutaneous injections. **D** Neoplasms removed from the sacrificed mice are shown from large to small according to volume. *Scale bars*: 1 cm. **E** Colony formation analysis of engineered CT26 cells in vitro. The error bars indicate the SDs (*P <  0.05, *n* = 3 independent experiments).

**Additional file 13: Figure S3.** MYSM1 is a favorable biomarker in several tumor patients. **A** Kaplan-Meier survival curve analysis of patients divided by MYSM1 expression in different tumors based on data from The Human Protein Atlas. The *P* log-rank test was used to determine statistical significance. **B** Table summarizing the results of statistical analysis of the 5-year survival rate based on data from The Human Protein Atlas. **C** Kaplan-Meier survival curve of OS based on the MYSM1 expression levels in breast cancer patients from the GEO database (GSE42568). Statistical significance was analyzed by the *P* log-rank test. **D** Kaplan-Meier analysis of the OS of patients with different stages of CRC (stages II-III) (GSE24551) according to their MYSM1 expression levels. The *P* log-rank test was used to evaluate statistical significance.

**Additional file 14: Figure S4.** MYSM1 inhibits the proliferative and metastatic capacities of CRC cells in vitro. **A** Growth curve analysis of the proliferation of Mysm1-knockout (KO)/control CT26 cells, siMYSM1−/siNC-transfected SW480 cells and MYSM1-overexpressing/control LOVO cells. The error bars indicate the SDs (*P <  0.05, *n* = 6 independent experiments). **B** and **C** Analysis of the cell cycle (**B**) and apoptosis (**C**) in siMYSM1−/siNC-transfected SW480 cells (*upper*) and MYSM1-overexpressing/control LOVO cells (*lower*) by flow cytometry in vitro. **D** and **E** The metastatic capacity of Mysm1-KO/control CT26 cells, siMYSM1−/siNC-transfected SW480 cells and MYSM1-overexpressing/control LOVO cells was measured by wound healing (**D**) and Transwell (**E**) assays. *Scale bars*: 50 μm. The data in **B-E** are presented as the means ± SDs (*P <  0.05 and **P <  0.01, *n* = 3 independent experiments). **F** Representative graphs of typical lesions in the livers of athymic BALB/c mice that received tail vein injections of Lenti-MYSM1−/Lenti-NC-infected SW620 cells.

**Additional file 15: Figure S5.** MYSM1 promotes the expression of miR-200 family members and inhibits the EMT process in CRC. **A** and **B** qRT-PCR analysis of the detailed alterations in the expression levels of 36 target miRNAs following MYSM1 downregulation in HCT116 cells (**A**) or MYSM1 upregulation in SW620 cells (**B**). The error bars represent the SDs (*P <  0.05, **P < 0.01 and ***P < 0.001, *n* = 3 independent experiments). **C** The mRNA levels of miR-200b, miR-200a, miR-429, miR-200c and miR-141 were measured by qRT-PCR in HCT116, SW480 and SW620 cells. The dotted line represents the normalized value of 1. The data are presented as the means ± SDs **D** The endogenous levels of MYSM1, CDH1 and vimentin in HCT116, SW480 and SW620 cells were examined by qRT-PCR (*left*) and western blot (*right*). The data are expressed as the means ± SDs.

**Additional file 16: Figure S6.** Pattern diagrams of the ChIP primers and restoration assay detection. **A** Locations and amplified fragments of the ChIP primers for the promoter regions of miR-200 family members and CDH1. The promoter regions of miR-200b cluster (chromosome 1, base pairs 0 ~ − 1500), miR-200c cluster (chromosome 12, base pairs 0 ~ − 2000) and CDH1 (chromosome 16, base pairs − 500 ~ + 1000) are shown. **B** The total protein expression of H2AK119ub1 was analyzed via western blot in HCT116 cells with MYSM1 knockdown and SW620 cells with MYSM1 overexpression. **C** and **D** qRT-PCR analysis of miR-200b and miR-200c levels in HCT116 (**C**) and SW620 (**D**) cells transfected with mimics/inhibitors compared with cells transfected with mimic/inhibitor NCs. The data are presented as the means ± SDs (**P < 0.01 and ***P < 0.001, *n* = 3 independent experiments). **E** Western blot analysis showing the rescuing effects of rapamycin (10 nmol/L) and wortmannin (100 nmol/L) on p-AKT (Ser473) and p-AKT (Thr308) in the context of MYSM1 knockdown-mediated PI3K/AKT signaling activation.

**Additional file 17: Figure S7.** MYSM1 restrains the progression of EMT in CRC cells by inducing the activation of miR-200 family members. HCT116 cells were transiently transfected with siNC, siMYSM1, mimic NC, miR-200b or miR-200c mimic as indicated; SW620 cells were transiently transfected with the control, pMSCV-MYSM1 vector, inhibitor NC, miR-200b or miR-200c inhibitor as indicated. **A-D** The proliferation ability of the transfected HCT116 (*left*) and SW620 (*right*) cells was measured by growth curve (**A** and **B**) and colony formation (**C** and **D**) assays, respectively. **E** and **F** Transwell assays were used to measure migration in HCT116 (**E**) and SW620 (**F**) cells transfected with the indicated constructs. *Scale bars:* 50 μm. The data are shown as the means ± SDs (*P < 0.05 and **P < 0.01, *n* = 3 independent experiments).

**Additional file 18: Figure S8.** MYSM1 is significantly suppressed in tumor tissues and is located mainly in the nucleus rather than the cytoplasm. **A** qRT-PCR analysis of the MYSM1 mRNA levels in seven paired patient specimens with kidney tumors (T) and adjacent normal tissues (N). **B** qRT-PCR analysis of the MYSM1 mRNA levels in 30 unpaired patient specimens, including 28 gliomas (T) and two adjacent normal tissues (N). **C** Scatter plot showing the distribution of MYSM1 expression in normal and tumor tissues in the kidneys (*left*) and brain (*right*). The data (**A-C**) are presented as the means ± SDs (*P < 0.05 and **P < 0.01, *n* = 3 independent experiments). **D** ROC curve analysis was used to distinguish MYSM1 expression in primary tumors (*left*) and metastatic tumors (*right*) of CRC patients from that in tissues of healthy individuals. The data are shown as the AUC values. **E** Comparison of two MYSM1 antibodies (Abcam #ab180570, Sigma-Aldrich #HPA054291) and identification of MYSM1 localization by IHC. *Scale bars*: 200 μm (*low power*), 50 μm (*high power*).

## Data Availability

All data generated or analyzed during this study are included in this published article and its additional files. The datasets generated and/or analyzed during the current study are available at the following hyperlinks: **1. Gene expression profiling interactive analysis** (GEPIA, http://gepia.cancer-pku.cn) **2. The human protein atlas** (https://www.proteinatlas.org) **3. Gene expression omnibus** (GEO, https://www.ncbi.nlm.nih.gov/geo/)

## Abbreviations

BAP1: Breast cancer type 1 susceptibility protein (BRCA1)-associated protein 1
BMI1: B-lymphoma Moloney murine leukemia virus insertion region 1 homolog
ChIP: Chromatin immunoprecipitation
CRC: Colorectal cancer
DUBs: Deubiquitinases
EMT: Epithelial-mesenchymal transition
H3K27me3: Trimethylated histone H3 at lysine 27
H3K4me3: Trimethylated histone H3 at lysine 4
HSC: Hematopoietic stem cell
IHC: Immunohistochemical staining
miRNAs: microRNAs
mRNA: messenger RNA
MYSM1: Myb-like, SWIRM and MPN domains 1
OS: Overall survival
qRT-PCR: Quantitative real-time polymerase chain reaction
RING1A: Ring finger protein 1A
RING1B: Ring finger protein 1B
TGF-β: Transforming growth factor-β
