# Supplementary material for: MYSM1 inhibits human colorectal cancer tumorigenesis by activating miR-200 family members/CDH1 and blocking PI3K/AKT signaling
Source: J Exp Clin Cancer Res. 2021 Oct 27;40:341. doi: 10.1186/s13046-021-02106-2 (PMC8549173; doi:10.1186/s13046-021-02106-2)
Supplement: Supplementary file 1 — Additional file 1: Table S1. Information of CRC patients collected for clinical studies. [file 13046_2021_2106_MOESM1_ESM.pdf]

Additional file 1

Table S1. Information of CRC patients collected for clinical studies

Patient Number	ID Number	Gender	Age	AJCC Pathologic TNM	Collected Tissue Specimens[a]
1	D201103233	Female	61	T4aN2b	N, T, L
2	D201109272	Male	62	T3N2b	N, T, L
3	D201104503	Male	59	T3N2b	N, T, L
4	D201105202	Female	43	T3N1b	N, T, L
5	D201002192	Female	75	T3N2Mx	N, T, L
6	D201002697	Female	44	T3N2Mx	N, T, L
7	D201002527	Female	69	T3N1Mx	N, T, L
8	D201311643	Male	66	T3N2a	N, T, L
9	D201308052	Female	78	T3N2a	N, T, L
10	D201302608	Female	65	T4aN2aM1a	N, T, H
11	D201308960	Male	50	T4aN1bM1	N, T, H
12	D201205252<br>D201201687	Male	69	T3N0Mx	N, T, H
13	D201503400	Male	38	T4bN1a	N, T, L
14	D201502630	Male	71	T4bN1a	N, T, L
15	D201501406	Female	27	T4aN1bM1a	N, T, L
16	D201412114	Male	66	T3N1a	N, T, L
17	D2014118161	Male	58	T3N1a	N, T, L
18	D201411260	Male	75	T4aN2b	N, T, L
19	D201411204	Female	50	T3N1b	N, T, L
20	D201410386	Male	53	T4aN2a	N, T, L

21	D201410268	Male	25	T4aN1a	N, T, L
22	D201408938	Male	52	T3N1b	N, T, L
23	D201406353	Male	23	T4aN2b	N, T, L
24	D201405498	Female	54	T3N1a	N, T, L
25	D201402883	Male	40	T3N2a	N, T, L
26	D201401103	Female	53	T3N2b	N, T, L
27	D201308257	Female	39	T4bN1bM1	N, T, L
28	D201300734	Female	71	T3N1a	N, T, L
29	D201300516	Male	55	T3N2b	N, T, L
30	D201204380	Male	74	T4aN1a	N, T, L
31	D201201553	Male	50	T4bN1b	N, T, L
32	D201103003	Female	59	T3N1cM1	N, T, H
33	D201002869	Female	76	T4N1Mx	N, T, L
34	D201000430	Male	48	T4N2M1	N, T, H
35	D201308206<br>D201506178	Female	56	T4aN0	N, T, L
36	D201302048<br>D201501948	Female	61<br>63	T3N1c	N, T, H
37	D201000011<br>D201410598	Male	57<br>61	T3N0Mx	N, T, H
38	200929911	Female	62	T3N2Mx	N, T, L
39	201002018	Female	53	T4N1Mx	N, T, L
40	200822027	Female	47	T3N2b	N, T, L
41	201210167<br>201205979	Male	62	T3N0	N, T, H

[a]N: adjacent normal tissue, T: primary tumor tissue, L: metastatic lymph node tumor tissue, H: metastatic hepatic tumor tissue.
