# Supplementary material for: MYSM1 inhibits human colorectal cancer tumorigenesis by activating miR-200 family members/CDH1 and blocking PI3K/AKT signaling
Source: J Exp Clin Cancer Res. 2021 Oct 27;40:341. doi: 10.1186/s13046-021-02106-2 (PMC8549173; doi:10.1186/s13046-021-02106-2)
Supplement: Supplementary file 2 — Additional file 2: Table S2. Information of CRC patients collected in tissue microarray of metastasis. [file 13046_2021_2106_MOESM2_ESM.pdf]

Additional file 2

Table S2. Information of CRC patients collected in tissue microarray of metastasis

Number	Age	Gender[a]	Organ	Pathological Diagnosis	Grade	Stage	TNM
1	55	M	Colon	Adenocarcinoma	2	IIIB	T3N1M0
2	55	M	Lymph node	Lymph node metastatic carcinoma	–	–	–
3	73	F	Colon	Adjacent normal colon tissue	–	–	–
4	39	M	Colon	Adenocarcinoma	1	IIIC	T4N2M0
5	39	M	Lymph node	Lymph node metastatic carcinoma	–	–	–
6	60	M	Colon	Adjacent normal colon tissue	–	–	–
7	71	M	Colon	Adenocarcinoma	1	IIIC	T4N2M0
8	71	M	Lymph node	Lymph node metastatic carcinoma	–	–	–
9	69	M	Colon	Adjacent normal colon tissue	–	–	–
10	65	M	Colon	Adenocarcinoma	1	IIIB	T3N1M0
11	65	M	Lymph node	Lymph node metastatic carcinoma	–	–	–
12	65	M	Colon	Adjacent normal colon tissue	–	–	–
13	60	M	Colon	Adenocarcinoma	1	IIIB	T3N1M0
14	60	M	Lymph node	Lymph node metastatic carcinoma	–	–	–
15	60	M	Colon	Adjacent normal colon tissue	–	–	–
16	57	M	Colon	Adenocarcinoma	1	IIIB	T3N1M0
17	57	M	Lymph node	Lymph node metastatic carcinoma	–	–	–
18	57	M	Colon	Adjacent normal colon tissue	–	–	–
19	58	M	Colon	Adenocarcinoma	2	IIIB	T3N1M0
20	58	M	Lymph node	Lymph node metastatic carcinoma	–	–	–
21	58	M	Colon	Adjacent normal colon tissue	–	–	–
22	65	M	Colon	Adenocarcinoma	2	IIIC	T4N2M0
23	47	M	Lymph node	Lymph node metastatic carcinoma	–	–	–
24	60	M	Colon	Adjacent normal colon tissue	–	–	–
25	64	F	Colon	Adenocarcinoma	3	IIIC	T4N2M0
26	65	M	Lymph node	Lymph node metastatic carcinoma	–	–	–
27	65	M	Colon	Adjacent normal colon tissue	–	–	–
28	52	F	Colon	Adenocarcinoma	2	IIIB	T3N2M0
29	52	F	Lymph node	Lymph node metastatic carcinoma	–	–	–

30	54	M	Colon	Adjacent normal colon tissue	–	–	–
31	58	M	Colon	Adenocarcinoma	3	IIIB	T3N1M0
32	58	M	Lymph node	Lymph node metastatic carcinoma	–	–	–
33	58	M	Colon	Adjacent normal colon tissue	–	–	–
34	69	M	Colon	Adenocarcinoma	2	IIIB	T3N1M0
35	69	M	Lymph node	Lymph node metastatic carcinoma	–	–	–
36	69	M	Colon	Adjacent normal colon tissue	–	–	–
37	57	F	Colon	Adenocarcinoma	2	IIIB	T3N2M0
38	57	F	Lymph node	Lymph node metastatic carcinoma	–	–	–
39	55	F	Colon	Adjacent normal colon tissue	–	–	–
40	30	M	Colon	Adenocarcinoma	2	IIIB	T3N1M0
41	30	M	Lymph node	Lymph node metastatic carcinoma	–	–	–
42	30	M	Colon	Adjacent normal colon tissue	–	–	–
43	70	M	Colon	Adenocarcinoma	3	IIIB	T3N1M0
44	70	M	Lymph node	Lymph node metastatic carcinoma	–	–	–
45	54	F	Colon	Adjacent normal colon tissue	–	–	–
46	56	M	Colon	Adenocarcinoma	2	IIIB	T3N1M0
47	56	M	Lymph node	Lymph node metastatic carcinoma	–	–	–
48	56	M	Colon	Adjacent normal colon tissue	–	–	–
49	49	F	Colon	Adenocarcinoma	3	IIIB	T3N2M0
50	49	F	Lymph node	Lymph node metastatic carcinoma	–	–	–
51	49	F	Colon	Adjacent normal colon tissue	–	–	–
52	70	M	Colon	Adenocarcinoma	3	IIIB	T3N2M0
53	70	M	Lymph node	Lymph node metastatic carcinoma	–	–	–
54	70	M	Colon	Adjacent normal colon tissue	–	–	–
55	42	M	Colon	Adenocarcinoma	3	IIIB	T3N1M0
56	42	M	Lymph node	Lymph node metastatic carcinoma	–	–	–
57	42	M	Colon	Adjacent normal colon tissue	–	–	–
58	63	M	Colon	Adenocarcinoma	3	IIIB	T3N1M0
59	63	M	Lymph node	Lymph node metastatic carcinoma	–	–	–
60	63	M	Colon	Adjacent normal colon tissue	–	–	–
61	47	F	Colon	Adenocarcinoma	3	IV	T4N2M0
62	64	F	Lymph node	Lymph node metastatic carcinoma	–	–	–
63	64	F	Colon	Adjacent normal colon tissue	–	–	–
64	60	F	Colon	Adenocarcinoma	2–3	IIIB	T3N1M0

65	60	F	Lymph node	Lymph node metastatic carcinoma	–	–	–
66	60	F	Colon	Adjacent normal colon tissue	–	–	–
67	56	F	Colon	Mucinous adenocarcinoma	2	IIIB	T4N1M0
68	56	F	Lymph node	Lymph node metastatic carcinoma	–	–	–
69	56	F	Colon	Adjacent normal colon tissue	–	–	–
70	72	F	Colon	Mucinous adenocarcinoma	2	IIIB	T3N1M0
71	72	F	Lymph node	Lymph node metastatic carcinoma	–	–	–
72	71	M	Colon	Adjacent normal colon tissue	–	–	–
73	30	M	Colon	Mucinous adenocarcinoma	3	IIIB	T3N2M0
74	30	M	Lymph node	Lymph node metastatic carcinoma	–	–	–
75	30	M	Colon	Adjacent normal colon tissue	–	–	–
76	43	M	Rectum	Adenocarcinoma	1	IIIB	T3N2M0
77	43	M	Lymph node	Lymph node metastatic carcinoma	–	–	–
78	43	M	Rectum	Adjacent normal rectum tissue	–	–	–
79	63	M	Rectum	Adenocarcinoma	2	IIIB	T3N2M0
80	63	M	Lymph node	Lymph node metastatic carcinoma	–	–	–
81	63	M	Rectum	Adjacent normal rectum tissue	–	–	–
82	56	M	Rectum	Adenocarcinoma	3	IIIB	T3N1M0
83	56	M	Lymph node	Lymph node metastatic carcinoma	–	–	–
84	56	M	Rectum	Adjacent normal rectum tissue	–	–	–
85	51	M	Rectum	Adenocarcinoma	3	IIIB	T3N1M0
86	51	M	Lymph node	Lymph node metastatic carcinoma	–	–	–
87	51	M	Rectum	Adjacent normal rectum tissue	–	–	–
88	44	F	Rectum	Adenocarcinoma	3	IIIB	T3N1M0
89	44	F	Lymph node	Lymph node metastatic carcinoma	–	–	–
90	44	F	Rectum	Adjacent normal rectum tissue	–	–	–
91	51	M	Rectum	Adenocarcinoma	3	IIIB	T3N1M0
92	51	M	Lymph node	Lymph node metastatic carcinoma	–	–	–
93	51	M	Rectum	Adjacent normal rectum tissue	–	–	–
94	47	M	Rectum	Mucinous adenocarcinoma	3	IIIB	T3N1M0
95	47	M	Lymph node	Lymph node metastatic carcinoma	–	–	–
96	48	F	Rectum	Adjacent normal rectum tissue	–	–	–
97	34	M	Rectum	Mucinous adenocarcinoma (sparse)	3	IIIB	T3N2M0
98	34	M	Lymph node	Lymph node metastatic carcinoma	–	–	–
99	34	M	Rectum	Adjacent normal rectum tissue	–	–	–

100	58	M	Colon	Adenocarcinoma	1	IV	T4N1M1
101	27	M	Colon	Adenocarcinoma	2	IIB	T4N0M0
102	35	M	Colon	Adenocarcinoma	2	III	T4N1M0
103	44	M	Colon	Adenocarcinoma	2	IIIC	T4N1M0
104	65	F	Colon	Adenocarcinoma	2	IIA	T3N0M0
105	52	F	Colon	Adenocarcinoma	1	IIA	T3N0M0
106	44	M	Colon	Adenocarcinoma	2	IIB	T4N0M0
107	33	F	Colon	Adenocarcinoma	1	IIB	T4N0M0
108	39	M	Colon	Adenocarcinoma	1	IIB	T4N0M0
109	66	M	Colon	Adenocarcinoma	2	IIA	T3N0M0
110	72	F	Colon	Adenocarcinoma	2	IIIC	T4N1M0
111	50	M	Colon	Adenocarcinoma	2	II	T4N0M0
112	75	M	Colon	Adenocarcinoma	2	IIB	T4N0M0
113	61	M	Colon	Adenocarcinoma	2	IIB	T4N0M0
114	53	M	Colon	Adenocarcinoma	2	IIA	T3N0M0
115	76	F	Colon	Adenocarcinoma	–	I	T2N0M0
116	70	M	Colon	Adenocarcinoma	2	I	T2N0M0
117	48	M	Colon	Adenocarcinoma	2	III	T2N1M0
118	43	F	Colon	Adenocarcinoma	3	III	T3N1M0
119	47	M	Colon	Adenocarcinoma	2	IIIC	T4N1M0
120	30	M	Colon	Adjacent normal colon tissue	–	–	–
121	33	M	Colon	Adjacent normal colon tissue	–	–	–
122	28	M	Colon	Adjacent normal colon tissue	–	–	–
123	30	M	Colon	Adjacent normal colon tissue	–	–	–
124	38	M	Colon	Adjacent normal colon tissue	–	–	–

1 [a]M: Male, F: Female
