# Supplementary material for: MYSM1 inhibits human colorectal cancer tumorigenesis by activating miR-200 family members/CDH1 and blocking PI3K/AKT signaling
Source: J Exp Clin Cancer Res. 2021 Oct 27;40:341. doi: 10.1186/s13046-021-02106-2 (PMC8549173; doi:10.1186/s13046-021-02106-2)
Supplement: Supplementary file 3 — Additional file 3: Table S3. Information of CRC patients collected in tissue microarray of survival. [file 13046_2021_2106_MOESM3_ESM.pdf]

Additional file 3

Table S3. Information of CRC patients collected in tissue microarray of survival

Patient Number	Histologic Type[a]	Survival State[b]	Overall Survival (Days)	Gender	Age	Pathological Diagnosis	Grade	T	N	M	AJCC Stage
1	T/N	0	2910	M	62	Tubular Adenocarcinoma	II	T3	N0	M0	2A
2	T/N	1	1200	M	75	Mucinous Adenocarcinoma	III	T4b	N0	M0	2C
3	T/N	0	2910	F	81	Tubular Adenocarcinoma	II	T3	N0	M0	2A
4	T/N	1	930	M	70	Mucinous Adenocarcinoma	II	T3	N0	M0	2A
5	T/N	1	90	F	73	Mucinous Adenocarcinoma	II-III	T3	N1b	M1b	4B
6	T/N	0	2880	M	71	Tubular Adenocarcinoma	II	T3	N0	M0	2A
7	T/N	1	1650	F	70	Tubular Adenocarcinoma	II-III	T3	N1a	M0	3B
8	T/N	0	2880	M	55	Tubular Adenocarcinoma	II-III	T3	N0	M0	2A
9	T/N	1	900	F	58	Tubular Adenocarcinoma	II-III	T3	N2a	M0	3B
10	T/N	1	150	M	72	Mucinous Adenocarcinoma	I-III	T3	N0	M0	2A
11	T/N	0	2850	F	62	Tubular Adenocarcinoma	II-III	T3	N0	M0	2A
12	T/N	1	1860	F	75	Tubular Adenocarcinoma	II	T3	N1b	M0	3B
13	T/N	1	1650	M	80	Tubular Adenocarcinoma	II	T3	N0	M0	2
14	T/N	1	720	M	75	Adenocarcinoma	II	T4a	N1b	M0	3B
15	T/N	0	2850	F	59	Tubular Adenocarcinoma	II-III	T3	N0	M0	2A
16	T/N	1	780	M	83	Adenocarcinoma	I-II	T4b	N1b	M0	3C
17	T/N	1	750	F	52	Tubular Adenocarcinoma	II	T3	N2a	M0	3B
18	T/N	0	2850	M	80	Tubular Adenocarcinoma	II-III	T3	N0	M0	2A

19	T/N	1	1680	M	81	Tubular Adenocarcinoma	Ⅰ-Ⅲ	T3	N0	M0	2A
20	T/N	1	1020	M	75	Tubular Adenocarcinoma	Ⅲ	T3	N0	M0	2A
21	T/N	1	390	M	75	Tubular Adenocarcinoma	Ⅱ-Ⅲ	T3	N1b	M0	3B
22	T/N	0	2820	M	–	Adenocarcinoma	Ⅲ	T3	N0	M0	2A
23	T/N	1	360	F	90	Tubular Adenocarcinoma	Ⅱ	T3	N0	M0	2A
24	T/N	0	2820	M	56	Tubular Adenocarcinoma	Ⅱ	T3	N0	M0	2A
25	T/N	0	2820	M	65	Adenocarcinoma	Ⅱ	T3	N0	M0	2A
26	T/N	0	2820	F	65	Adenocarcinoma	Ⅲ	–	N1b	M0	3
27	T/N	1	240	M	71	Tubular Adenocarcinoma	Ⅱ-Ⅲ	T3	N1b	M1b	4B
28	T/N	1	840	F	76	Tubular Adenocarcinoma	Ⅰ-Ⅲ	T4b	N1a	M0	3C
29	T/N	0	2790	M	72	Tubular Adenocarcinoma	Ⅱ	T4a	N0	M0	2B
30	T/N	1	1560	M	76	Tubular Adenocarcinoma	Ⅱ-Ⅲ	T3	N0	M0	2A
31	T/N	1	390	–	72	Tubular Adenocarcinoma	Ⅱ-Ⅲ	T3	N2a	M1b	4B
32	T/N	1	780	M	38	Adenocarcinoma	Ⅰ-Ⅲ	T4a	N0	M0	2B
33	T/N	0	2790	M	–	Adenocarcinoma	Ⅱ	T3	N0	M0	2A
34	T/N	1	2370	F	65	Tubular Adenocarcinoma	Ⅰ-Ⅲ	T4a	N0	M0	2B
35	T/N	0	2790	M	79	Adenocarcinoma	Ⅱ	T3	N0	M0	2A
36	T/N	1	600	M	75	Mucinous Adenocarcinoma	Ⅲ	–	N1a	M0	3
37	T/N	1	30	F	74	Adenocarcinoma	Ⅱ	T4b	N2a	M0	3C
38	T/N	1	1200	M	50	Adenocarcinoma	Ⅰ	T3	N0	M0	2A
39	T/N	0	2760	F	82	Adenocarcinoma	Ⅱ	T3	N1b	M0	3B
40	T/N	1	2610	F	56	Mucinous Adenocarcinoma	Ⅱ	T4a	N0	M0	2B
41	T/N	0	2760	F	83	Adenocarcinoma	Ⅰ	T2	N0	M0	1
42	T/N	0	2760	M	72	Adenocarcinoma	Ⅲ	T3	N0	M0	2A
43	T/N	1	840	F	89	Mucinous Adenocarcinoma	Ⅲ	T3	N0	M0	2A
44	T/N	1	690	M	60	Mucinous Adenocarcinoma	Ⅰ-Ⅲ	T3	–	M0	–
45	T/N	1	2160	M	78	Tubular	Ⅱ-Ⅲ	T3	N1a	M0	3B

						Adenocarcinoma					
46	T/N	1	180	M	80	Tubular Adenocarcinoma	Ⅲ	T4b	N0	M0	2C
47	T/N	1	1500	M	62	Adenocarcinoma	Ⅱ	T3	N0	M0	2A
48	T/N	1	480	M	–	Tubular Adenocarcinoma	Ⅱ-Ⅲ	T3	N0	M0	2A
49	T/N	0	2730	F	–	Tubular Adenocarcinoma	Ⅱ-Ⅲ	T3	N0	M0	2A
50	T/N	1	540	F	71	Tubular Adenocarcinoma	Ⅱ-Ⅲ	T2	N0	M0	1
51	T/N	0	2730	F	70	Adenocarcinoma	Ⅱ	T3	N1b	M0	3B
52	T/N	1	600	M	74	Adenocarcinoma	Ⅱ	T2	N1a	M0	3A
53	T/N	1	480	F	79	Adenocarcinoma	Ⅱ	T4b	N1b	M0	3C
54	T/N	1	1560	F	80	Adenocarcinoma	Ⅱ	T3	N0	M0	2A
55	T/N	1	1080	M	90	Tubular Adenocarcinoma	Ⅱ-Ⅲ	T3	N0	M0	2A
56	T/N	1	150	M	48	Adenocarcinoma	Ⅲ	T4b	N0	M0	2C
57	T/N	1	300	F	67	Adenocarcinoma	Ⅱ	T4a	N1a	M0	3B
58	T/N	1	420	F	84	Mucinous Adenocarcinoma	Ⅰ-Ⅲ	T3	N1a	M0	3B
59	T/N	0	2670	F	57	Tubular Adenocarcinoma	Ⅱ-Ⅲ	T2	N0	M0	1
60	T/N	0	2670	M	65	Adenocarcinoma	Ⅱ	T3	N0	M0	2A
61	T/N	1	1860	M	80	Mucinous Adenocarcinoma	Ⅰ-Ⅱ	T3	N0	M0	2A
62	T/N	1	870	M	67	Tubular Adenocarcinoma	Ⅱ-Ⅲ	T3	N1b	M0	3B
63	T/N	1	870	M	72	Adenocarcinoma	Ⅱ	T3	N1a	M0	3B
64	T/N	0	2670	M	62	Tubular Adenocarcinoma	Ⅱ	T3	N0	M0	2A
65	T/N	0	2640	F	57	Tubular Adenocarcinoma	Ⅱ-Ⅲ	T3	N1a	M0	3B
66	T/N	0	2640	F	50	Adenocarcinoma	Ⅱ-Ⅲ	T3	N0	M0	2A
67	T/N	0	2640	M	78	Adenocarcinoma	Ⅰ-Ⅱ	T1	N0	M0	1
68	T/N	0	2640	F	77	Adenocarcinoma	Ⅱ	T3	N0	M0	2A
69	T/N	0	2640	F	84	Adenocarcinoma	Ⅱ	T2	N0	M0	1
70	T/N	0	2640	M	80	Adenocarcinoma	Ⅲ	T3	N1b	M0	3B
71	T/N	0	2640	F	65	Adenocarcinoma	Ⅱ	T3	N0	M0	2A
72	T/N	1	2460	M	74	Adenocarcinoma	Ⅱ	T3	N0	M0	2A
73	T/N	0	2640	F	65	Adenocarcinoma	Ⅱ	T3	N2a	M0	3B
74	T/N	1	1680	M	74	Adenocarcinoma	Ⅰ-Ⅱ	T3	N0	M0	2A
75	T/N	1	450	F	71	Mucinous	Ⅰ-Ⅲ	T3	N2a	M0	3B

						Adenocarcinoma					
76	T/N	1	720	F	67	Adenocarcinoma	Ⅱ-Ⅲ	T3	N1b	M0	3B
77	T/N	0	2640	M	58	Adenocarcinoma	Ⅰ-Ⅱ	T3	N0	M0	2A
78	T/N	1	2400	M	62	Adenocarcinoma	Ⅱ	T2	N0	M0	1
79	T/N	0	2610	F	54	Tubular Adenocarcinoma	Ⅱ	T3	N0	M0	2A
80	T/N	1	330	M	76	Tubular Adenocarcinoma	Ⅱ	T3	N0	M0	2A
81	T	0	2880	F	70	Mucinous Adenocarcinoma	Ⅱ	T3	N0	M0	2A
82	T	1	540	M	48	Tubular Adenocarcinoma	Ⅱ-Ⅲ	T3	N2a	M0	3B
83	T	1	1530	F	24	Tubular Adenocarcinoma	Ⅱ-Ⅲ	T3	N2a	M0	3B
84	T	1	150	F	73	Mucinous Adenocarcinoma	Ⅲ	T3	N2a	M0	3B
85	T	0	2820	M	74	Adenocarcinoma	Ⅱ	–	N1a	M0	3
86	T	1	270	F	68	Adenocarcinoma	Ⅲ	T3	N1b	M0	3B
87	T	1	390	M	42	–	Ⅲ	–	–	M0	–
88	T	0	2760	M	52	Mucinous Adenocarcinoma	Ⅱ-Ⅲ	T3	N0	M0	2A
89	T	1	360	F	62	Adenocarcinoma	Ⅲ	T3	N2b	M0	3C
90	T	0	2730	M	–	Tubular Adenocarcinoma	Ⅱ	T3	N0	M0	2A
91	T	1	2100	M	–	Adenocarcinoma	Ⅱ-Ⅲ	T3	N0	M0	2A
92	T	0	2700	M	62	Adenocarcinoma	Ⅲ	T3	N0	M0	2A
93	T	1	630	F	62	Adenocarcinoma	Ⅱ	T3	N2b	M0	3C
94	T	1	870	F	78	Adenocarcinoma	Ⅱ	T3	N1a	M0	3B
95	T	0	2670	F	79	Mucinous Adenocarcinoma	Ⅱ	T3	N1b	M0	3B
96	T	1	1020	F	76	Adenocarcinoma	Ⅱ	T3	N0	M0	2A
97	T	0	2640	F	54	Tubular Adenocarcinoma	Ⅱ-Ⅲ	T4a	N1a	M0	3B
98	T	1	1860	M	73	Tubular Adenocarcinoma	Ⅱ	T3	N0	M0	2A
99	T	1	180	F	73	Adenocarcinoma	Ⅱ	T3	N0	M0	2A
100	T	0	2610	M	53	Adenocarcinoma	Ⅱ-Ⅲ	T3	N0	M0	2A

[a]T: Tumor Tissues, N: Normal Tissues; [b]0: Survival, 1: Death
