# Supplementary material for: MYSM1 inhibits human colorectal cancer tumorigenesis by activating miR-200 family members/CDH1 and blocking PI3K/AKT signaling
Source: J Exp Clin Cancer Res. 2021 Oct 27;40:341. doi: 10.1186/s13046-021-02106-2 (PMC8549173; doi:10.1186/s13046-021-02106-2)
Supplement: Supplementary file 4 — Additional file 4: Table S4. Oligonucleotide sequences in this study. [file 13046_2021_2106_MOESM4_ESM.pdf]

Additional file 4

Table S4. Oligonucleotide sequences in this study

Oligonucleotides	Sense (5’-3’)	Antisense (5’-3’)
mimic NC/siNC	UUCUCCGAACGUGUCACGUTT	ACGUGACACGUUCGGAGAATT
miR-200b mimic	UAAUACUGCCUGGUAAUGAUGA	AUCAUUACCAGGCAGUAUUAUU
miR-200c mimic	UAAUACUGCCGGGUAAUGAUGGA	CAUCAUUACCCGGCAGUAUUAUU
inhibitor NC	CAGUACUUUUGUGUAGUACAA	
miR-200b inhibitor	UCAUCAUUACCAGGCAGUAUUA	
miR-200c inhibitor	UCCAUCAUUACCCGGCAGUAUUA	
siMYSM1 (si560)	CCGGCCAUAAUCUUCAAGUTT	ACUUGAAGAUUAUGGCCGGTT
siMYSM1 (silencer 1)	GGAGGUGCAAAGUUCAUUGTT	CAAUGAACUUUGCACCUCCTC
siMYSM1 (silencer 2)	CGGAUUCAUACAUACCUCGTT	CGAGGUAUGUAUGAAUCCGTC
siMYSM1 (silencer 3)	GCCGCACUGUUUUACAAGUTT	ACUUGUAAAACAGUGCGGCTT
