# Supplementary material for: MYSM1 inhibits human colorectal cancer tumorigenesis by activating miR-200 family members/CDH1 and blocking PI3K/AKT signaling
Source: J Exp Clin Cancer Res. 2021 Oct 27;40:341. doi: 10.1186/s13046-021-02106-2 (PMC8549173; doi:10.1186/s13046-021-02106-2)
Supplement: Supplementary file 5 — Additional file 5: Table S5. Antibodies used for ChIP, western blot, IHC and IF assays in this study. [file 13046_2021_2106_MOESM5_ESM.pdf]

Additional file 5

Table S5. Antibodies used for ChIP, western blot, IHC and IF assays in this study

Antigen	Species	Applications and Dilutions	Source
IgG	Rabbit	2μg in ChIP	Cell Signaling Technology #2729
MYSM1	Rabbit	WB (1:1000)	Abcam #ab107542
MYSM1	Rabbit	IHC (1:2000); 5μg in ChIP	Sigma-Aldrich #HPA054291
CDH1	Rabbit	WB (1:500); 5μg in ChIP	Santa Cruz Biotechnology #sc-7870
Vimentin	Mouse	WB (1:500)	Santa Cruz Biotechnology #sc-66002
PCNA	Rabbit	WB (1:1000); IF (1:800)	Cell Signaling Technology #13110
H2AK119ub1	Rabbit	WB (1:1000); 5μg in ChIP	Cell Signaling Technology #8240
H3K4me3	Rabbit	5μg in ChIP	Cell Signaling Technology #9751
H3K27me3	Rabbit	5μg in ChIP	Cell Signaling Technology #9733
p-AKT (Ser473)	Rabbit	WB (1:1000)	Cell Signaling Technology #4060
p-AKT (Thr308)	Rabbit	WB (1:1000)	Cell Signaling Technology #13038
p-PTEN (Ser380)	Rabbit	WB (1:1000)	Cell Signaling Technology #9551
AKT (pan)	Rabbit	WB (1:1000)	Cell Signaling Technology #4691
p-PDK1 (Ser241)	Rabbit	WB (1:1000)	Cell Signaling Technology #3438
p-GSK-3β (Ser9)	Rabbit	WB (1:1000)	Cell Signaling Technology #5558
β-actin	Mouse	WB (1:2000)	Sigma-Aldrich #A1978
GAPDH	Mouse	WB (1:2000)	Fantibody #FAB100064
Anti-rabbit IgG	Goat	WB (1:4000)	Cell Signaling Technology #7074
Anti-mouse IgG	Goat	WB (1:4000)	Jing Cai #PB002H
