# Supplementary material for: MYSM1 inhibits human colorectal cancer tumorigenesis by activating miR-200 family members/CDH1 and blocking PI3K/AKT signaling
Source: J Exp Clin Cancer Res. 2021 Oct 27;40:341. doi: 10.1186/s13046-021-02106-2 (PMC8549173; doi:10.1186/s13046-021-02106-2)
Supplement: Supplementary file 6 — Additional file 6: Table S6. Primer sequences for the 36 miRNAs in qRT-PCR assay of CRC cells. [file 13046_2021_2106_MOESM6_ESM.pdf]

Additional file 6

Table S6. Primer sequences for the 36 miRNAs in qRT-PCR assay of CRC cells

Primers for miRNAs	Forward (5'-3')[a]
hsa-miR-9	TCTTTGGTTATCTAGCTGTATGA
hsa-miR-17	CAAAGTGCTTACAGTGCAGGTAG
hsa-miR-132	TAACAGTCTACAGCCATGGTCG
hsa-miR-141	TAACACTGTCTGGTAAAGATGG
hsa-miR-182	TTTGGCAATGGTAGAACTCACACT
hsa-miR-191	CAACGGAATCCCAAAAGCAGCTG
hsa-miR-210	CTGTGCGTGTGACAGCGGCTGA
hsa-miR-345	GCTGACTCCTAGTCCAGGGCTC
hsa-miR-212	TAACAGTCTCCAGTCACGGCC
hsa-miR-429	GGTAATACTGTCTGGTAAAACCG
hsa-miR-484	TCAGGCTCAGTCCCCTCCCGAT
hsa-miR-125b	TCCCTGAGACCCTAACTTGTGA
hsa-miR-135b	TATGGCTTTTCATTCCTATGTGA
hsa-miR-145	GTCCAGTTTTCCCAGGAATCCC
hsa-miR-148a	TCAGTGCACTACAGAACTTTGT
hsa-miR-153	TTGCATAGTCACAAAAGTGATC
hsa-miR-18a	TAAGGTGCATCTAGTGCAGATAG
hsa-miR-193b	AACTGGCCCTCAAAGTCCCGCT
hsa-miR-19a	TGTGCAAATCTATGCAAAACTGA
hsa-miR-19b-1	AGTTTTGCAGGTTTGCATCCAGC
hsa-miR-200a	TAACACTGTCTGGTAACGATGT
hsa-miR-200b	TAATACTGCCTGGTAATGATGA
hsa-miR-200c	TAATACTGCCGGGTAATGATGGA

hsa-miR-23a	ATCACATTGCCAGGGATTTCC
hsa-miR-219a-1	AGAGTTGAGTCTGGACGTCCCG
hsa-miR-20a	TAAAGTGCTTATAGTGCAGGTAG
hsa-miR-24-2	TGCCTACTGAGCTGAAACACAG
hsa-miR-27a	TTCACAGTGGCTAAGTTCCGC
hsa-miR-365a	TAATGCCCCTAAAAATCCTTAT
hsa-miR-375	TTTGTTCGTTCGGCTCGCGTGA
hsa-miR-487b	AATCGTACAGGGTCATCCACTT
hsa-miR-92a-1	AGGTTGGGATCGGTTGCAATGCT
hsa-miR-92b	TATTGCACTCGTCCCGGCCTCC
hsa-let-7a-2	CTGTACAGCCTCCTAGCTTTCC
hsa-let-7d	AGAGGTAGTAGGTTGCATAGTT
hsa-let-7i	TGAGGTAGTAGTTTGTGCTGTT

[a]The RR716 (SYBR® PrimeScript™ miRNA RT-PCR Kit, TaKaRa) was used for miRNA Reverse Primers (5’-3’).
