# Supplementary material for: MYSM1 inhibits human colorectal cancer tumorigenesis by activating miR-200 family members/CDH1 and blocking PI3K/AKT signaling
Source: J Exp Clin Cancer Res. 2021 Oct 27;40:341. doi: 10.1186/s13046-021-02106-2 (PMC8549173; doi:10.1186/s13046-021-02106-2)
Supplement: Supplementary file 7 — Additional file 7: Table S7. Primer sequences for the miR-200 family members/CDH1 in ChIP assay. [file 13046_2021_2106_MOESM7_ESM.pdf]

Additional file 7

Table S7. Primer sequences for the miR-200 family members/CDH1 in ChIP assay

Sets of primers	Forward (5'-3')	Reverse (5'-3')
**miR-200b-a-429**		
1 (-1426 ~ -1240 bp)	CCAGGGGCTCCAAAGTAACC	CACAGCTGGGGGAATGAGG
2 (-1215 ~ -987 bp)	CGAGCCCCTGGCGAGGAG	GCCTTAGAAGCGGGTAGGGT
3 (-784 ~ -653 bp)	GGGCCTGCGTCACCGTCACT	GGGGCTCGCCTTACAAGGA
4 (-591 ~ -363 bp)	CGTGAAGAGCCGATGCTTTA	TCAATGTGCTGCCAACCA
5 (-160 ~ -13 bp)	GCTGCTCGTTGGCTTTACA	TCAGTCATTGCGTTCTCACC
**miR-200c-141**		
1 (-1967 ~ -1762 bp)	CTGGAGTGGAGCAAGCGATG	CTGTCGGGACCGCTGTG
2 (-1564 ~ -1408 bp)	CTTTTCGCCGAGACTGG	AGGGGCACTGAGGAGCATTG
3 (-1088 ~ -926 bp)	CGTCCCCCACTACAGTGTAA	TCGGAATTTGGAGGTATCGG
4 (-722 ~ -505 bp)	CCCCTTGTCCATACCTG	TGGCAAGGTCAACAGCTAAG
5 (-361 ~ -180 bp)	CCCTGGATCTTCCCGTCAGC	CAGGCAAGGGCGAGGAC
**CDH1**		
1 (-490 ~ -345 bp)	AATTAGCCTGGCGTGGTGGTGTG	GGGGTCTCACTCTTTCACCCAAGC
2 (+772 ~ +998 bp)	AGGTCTTGAGGGGGTGACTC	GGGGGAAAGGTAGATGGAAC
3 (+1309 ~ +1523 bp)	CCCTGCCTGGTTGTTGACTA	CTGACCCTGAGCAAGTGGAC
