# Supplementary material for: MYSM1 inhibits human colorectal cancer tumorigenesis by activating miR-200 family members/CDH1 and blocking PI3K/AKT signaling
Source: J Exp Clin Cancer Res. 2021 Oct 27;40:341. doi: 10.1186/s13046-021-02106-2 (PMC8549173; doi:10.1186/s13046-021-02106-2)
Supplement: Supplementary file 8 — Additional file 8: Table S8. Primers used for qRT-PCR analysis in this study. [file 13046_2021_2106_MOESM8_ESM.pdf]

Additional file 8

Table S8. Primers used for qRT-PCR analysis in this study

Gene	Forward (5'-3')	Reverse (5'-3')
MYSM1	CACAGGTACCCACATTGCTG	CTGTATCATAGGCCCCCTCA
CDH1	TGCCCAGAAAATGAAAAAGG	GTGTATGTGGCAATGCGTTC
Vimentin	GAGAACTTTGCCGTTGAAGC	GCTTCCTGTAGGTGGCAATC
FN1	CTGGCCGAAAATACATTGTAAA	CCACAGTCGGGTCAGGAG
ETS1	ACAGGGTAAGTGAAGGTTAATTCCA	AGAAAGATGACTACCTTGCTTGACT
SNAI1	ACAAGCACCAAGAGTCCG	ATGGCAGTGAGAAGGATGTG
ZEB1	GACAGTGTTACCAGGGAGGAGCA	TTCAGGTGCCTCAGGAAAAATGA
PTEN	CTCAGCCGTTACCTGTGTGT	GGTTTCCTCTGGTCCTGGT
PHLPP1	GCAGGAAAACCTCACAGCA	AGGATGACTTGGCGTCTTGT
U6	CTCGCTTCGGCAGCACA	AACGCTTCACGAATTTGCGT
β-actin	TGGCATCCACGAAACTACC	GTGTTGGCGTACAGGTCTT
