# Supplementary material for: MYSM1 inhibits human colorectal cancer tumorigenesis by activating miR-200 family members/CDH1 and blocking PI3K/AKT signaling
Source: J Exp Clin Cancer Res. 2021 Oct 27;40:341. doi: 10.1186/s13046-021-02106-2 (PMC8549173; doi:10.1186/s13046-021-02106-2)
Supplement: Supplementary file 9 — Additional file 9: Table S9. Primers used for construction of plasmids in CRISPR-Cas9 assay. [file 13046_2021_2106_MOESM9_ESM.pdf]

Additional file 9

Table S9. Primers used for construction of plasmids in CRISPR-Cas9 assay

Gene	Forward (5'-3')	Reverse (5'-3')
Mysm1 (1)	CACCGGCCGCTCGTACTTTATCAAG	AAACCTTGATAAAGTACGAGCGGCC
Mysm1 (2)	CACCGAAGCAGCATACCAGCTTGCC	AAACGGCAAGCTGGTATGCTGCTTC
