# Supplementary material for: MYSM1 inhibits human colorectal cancer tumorigenesis by activating miR-200 family members/CDH1 and blocking PI3K/AKT signaling
Source: J Exp Clin Cancer Res. 2021 Oct 27;40:341. doi: 10.1186/s13046-021-02106-2 (PMC8549173; doi:10.1186/s13046-021-02106-2)
Supplement: Supplementary file 10 — Additional file 10. Supplementary methods. [file 13046_2021_2106_MOESM10_ESM.pdf]

Additional file 10: Supplementary methods

Cell lines and cultivation

The CRC cell lines HCT116, SW480, SW620 and LOVO were obtained from the Cell Bank of Chinese Academy Sciences (SIBS, Shanghai, China). HCT116 cells were maintained in McCoy's 5A (modified) medium (Gibco, Los Angeles, USA). SW480 and SW620 cells were cultivated in Leibovitz′s L-15 medium (Gibco). LOVO cells were cultured in Roswell Park Memorial Institute medium-1640 (RPMI-1640, Gibco). All target cells were incubated at 37 °C in a 5 % CO_2 atmosphere, and all media were supplemented with 10 % fetal bovine serum (FBS) (Gibco) and 1 % penicillin-streptomycin. CRC cells were used for the *in vitro* and *in vivo* experiments.

Transient transfection

CRC cells were transiently transfected with plasmids or oligonucleotides as indicated via Lipofectamine® 2000 reagent (Invitrogen, Carlsbad, USA) at a final concentration of 50 nM for 48 h according to the manufacturer's instructions. The efficiency of the transient transfection was monitored by quantitative real-time polymerase chain reaction (qRT-PCR) and western blot analyses.

RNA isolation, reverse transcription reaction and qRT-PCR

Total RNA, including mRNA and miRNA, was extracted and purified from CRC tissue specimens via a miRNeasy Formalin-Fixed, Paraffin Embedded (FFPE) Kit

(Qiagen, Hilden, Germany) and from CRC cells with TRIzol reagent (Invitrogen). After extraction, each RNA sample was quantified by a Nanodrop 2000 instrument, and mRNA and miRNA reverse transcription reactions were performed with PrimeScript™ RT Master Mix and SYBR® PrimeScript™ miRNA RT-PCR Kit (TaKaRa, Shiga, Japan), respectively. The relative levels of mRNA and miRNA were quantified by FastStart Essential DNA Green Master (Roche, Indianapolis, USA) on a Bio-Rad CFX96 system (Bio-Rad, Hercules, USA) following the manufacturer's protocols. Normalization relative to the levels of β-actin and U6 was performed for the mRNA and miRNA analyses, respectively. A hot start PCR reaction was conducted according to the following protocol followed by a melting curve analysis: annealing at 95 °C for 10 s and extension at 58 °C for 30 s for 40 cycles. The fold changes relative to the controls (β-actin and U6) were calculated by the $^{\Delta\Delta}$CT method. All qRT-PCR experiments were performed in at least triplicate.

Oligonucleotides, lentivirus construction and stable infection

The oligonucleotides, including the microRNA mimics with the negative control (NC), microRNA inhibitors with the inhibitor negative control (inhibitor NC), siRNAs, silencers and the silencer negative control (siRNA NC), were synthesized by GenePharma (Shanghai, China). The MYSM1 coding sequence (CDS) was amplified by PCR and cloned into the expression plasmid pMSCV, thus creating an MYSM1 overexpression vector. The pMSCV-MYSM1 plasmid was used to build MYSM1

overexpression lentiviral particles by GeneCopoeia (Guangzhou, China). SW620 cells were stably infected with MYSM1-overexpressing or control lentiviral particles (Lenti-MYSM1 or Lenti-NC, respectively) according to the manufacturer's instructions. Polybrene (8 μg/mL) (Millipore, Billerica, USA) was added to each target cell line to help achieve the infection.

Protein isolation and western blot analysis

Whole cell lysates were isolated by RIPA lysis buffer (Jingcai, Shaanxi, China) supplemented with a protease inhibitor cocktail (Roche). The final concentration of the target proteins was quantified via a bicinchoninic assay (BCA). The samples of each cell lysate (25 μg) were separated by 10 % SDS-PAGE gel at a constant voltage and then transferred at a constant current onto polyvinylidene fluoride (PVDF) membranes (Millipore). The PVDF membranes were blocked in 5 % bovine serum albumin (BSA) diluted in TBST (Tris-buffered saline with 0.05 % Tween 20) for at least 1 h at room temperature. Then, the membranes were incubated with the appropriate primary antibodies overnight at 4 °C. The PVDF membranes were washed with TBST three times and incubated with secondary antibodies for 1 h at room temperature. The signal of the target band was detected by a FluorChem FC2 system (Alpha Innotech, San Leandro, USA) according to the manufacturer's instructions.

Growth curve assay

A tetrazolium salt 3-(4, 5-dimethylthiazol-2-yl)-2, 5-diphenyltetrazolium bromide (MTT) assay was used to detect cell proliferation *in vitro*. First, each group of target cells was transiently transfected with the appropriate oligonucleotides for 24 h before the MTT assay; then, the cells (2×10^3 cells per well) were plated in 96-well plates in 200 μL of medium supplemented with 10 % FBS. The MTT substrate (20 μL at 2.5 mg/mL in double distilled water) was added to each well, and the samples were incubated at 37 °C with 5 % CO_2 for an additional 4 h before the analysis. Then, the serum medium was removed, and 150 μL of dimethylsulfoxide (DMSO) was added to solubilize the cells at room temperature under slight rocking for 15 min. The single absorbance at 490 nm was monitored via colorimetric analysis by a microplate reader (Bio-Rad). The MTT assay was performed 0, 24, 48, 72, 96 and 120 h after transfection, and each experiment was performed in triplicate.

Colony formation assay

The experimental cells (2×10^3) were treated with the indicated oligonucleotides and plated in 6-cm dishes. The 6-cm dishes were filled with 10 mL of 10 % FBS medium. The cells were incubated at 37 °C with 5 % CO_2 for 10-14 days. Then, the cells were washed with PBS three times and fixed in methyl alcohol for 20 min at room temperature. The methyl alcohol was removed, and the colonies were stained with Giemsa for 15-20 min. The dye was discarded, and the plates were air dried.

Finally, the 6-cm dishes were inverted, and the visible colonies were counted.

Cell cycle and apoptosis analyses

The cell cycle and apoptosis were analyzed by flow cytometry. Briefly, for the cell cycle analysis, target cells with the appropriate transfections were harvested and washed thrice with ice-cold PBS. Then, the cell pellets were fixed with 70 % ethanol at 4 °C overnight. After centrifugation, the supernatant was removed, and the cells were resuspended in PBS with propidium iodide (PI) (40 μg/mL, BD Bioscience, San Jose, USA) and RNase (100 μg/mL) at 37 °C for 30 min. The cell cycle was analyzed by a FACScan flow cytometer (BD Bioscience). For the apoptosis analysis, the target cells were harvested, resuspended in PBS with FITC-conjugated Annexin V and PE-labeled PI and incubated at 4 °C for 30 min in the dark. Apoptosis was measured by an EPICS XL flow cytometer (Beckman Coulter, Pasadena, USA) at 488 nm and analyzed by CellQuest software. The experiments involving these two indexes were repeated at least three times.

Wound healing and Transwell assays

Cell migration was detected by wound-healing and Transwell assays. For the wound-healing assay, in total, 5×10^5 cells were plated in 6-well plates and cultivated until 90 % confluence was reached. A 100 μL pipette tip was used to scratch the monolayer, creating a wound. Wound closure was observed by taking photos under a

microscope 0, 24, 48, and 72 h after scratching. For the Transwell assay, 8-μm 24-well Transwell chambers (Millipore) were used according to the manufacturer's instructions. The cells (8×10^3) were appropriately pretreated and seeded in 200 μL of serum-free medium on the upper chamber, and 500 μL medium with 10 % FBS was added to the lower compartment as a chemoattractant. After a 48 h incubation, the migrated cells were fixed with absolute ethyl alcohol for 20 min and stained with 0.1 % crystal violet for 20 min at room temperature; then, five randomly selected fields were photographed. The cells in the fields were counted under a high-power microscope. Each experiment was performed in triplicate.

Immunofluorescence (IF)

SW620 cells stably infected with lentiviral particles were seeded on coverslips and cultured for 24 h. Then, the adherent cells were washed with PBS multiple times and fixed with 4 % paraformaldehyde for 20 min. Next, the cells were permeabilized with 0.1 % Triton X-100 before blocking with 5 % BSA for 2 h at room temperature. The target cells were incubated with the appropriate primary antibodies at 4 °C overnight. Then, the cells were incubated with Cy3-conjugated secondary antibodies for 1 h at room temperature in the dark. The supernatant was removed, and the cells were washed several times with PBS. The nuclei of the cells were stained with 4, 6-diamidino-2-phenylindole (DAPI) at a final concentration of 0.1 μg/mL. The images were visualized, and photos were taken under a fluorescence microscope.
