# Supplementary figures and images for: MYSM1 inhibits human colorectal cancer tumorigenesis by activating miR-200 family members/CDH1 and blocking PI3K/AKT signaling
Source: J Exp Clin Cancer Res. 2021 Oct 27;40:341. doi: 10.1186/s13046-021-02106-2 (PMC8549173; doi:10.1186/s13046-021-02106-2)

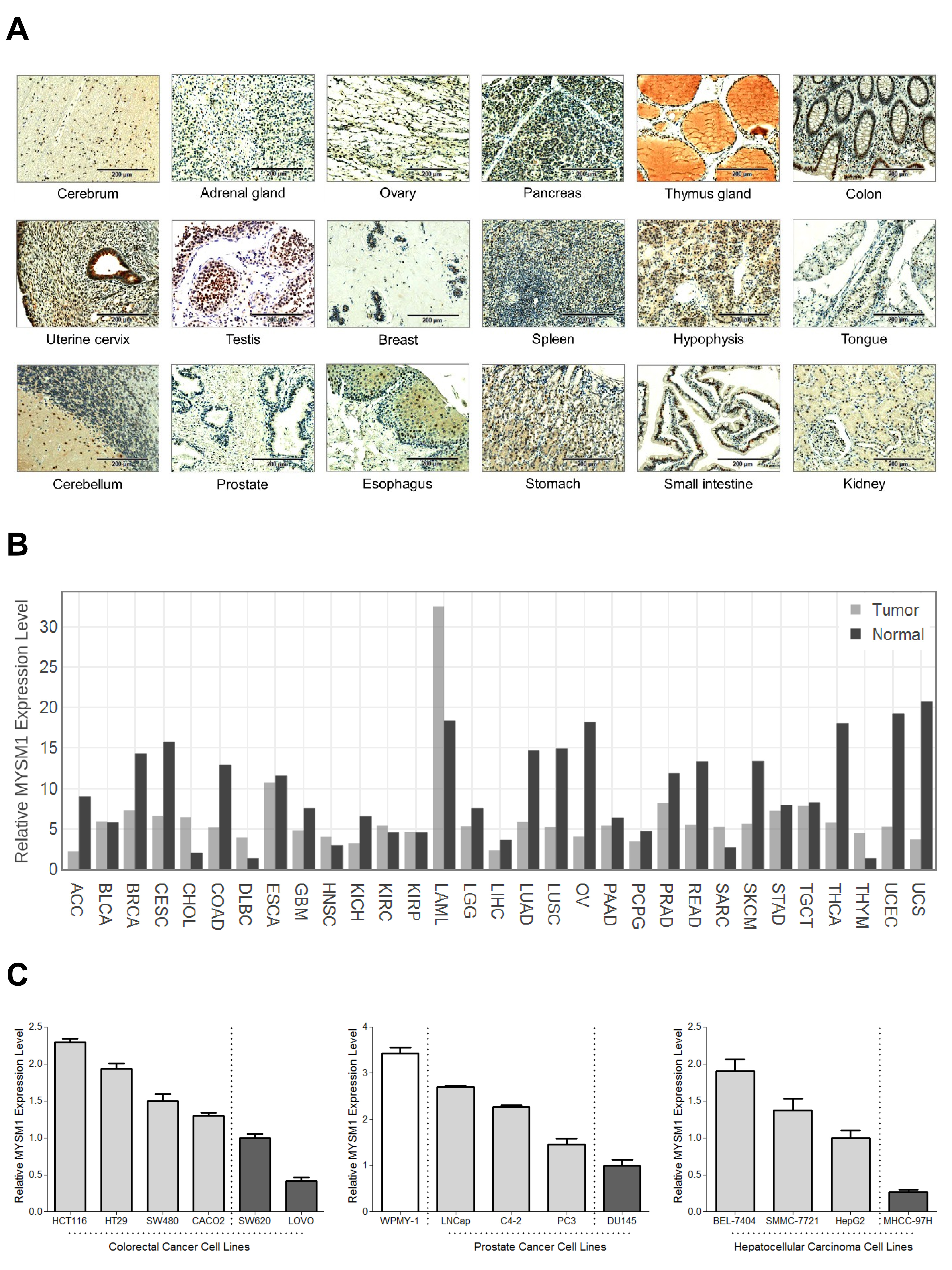

Supplement: Supplementary file 11 — Additional file 11: Figure S1. MYSM1 widely exists and is differentially expressed in normal vs. tumor tissues in the human body. A Representative IHC of MYSM1 in a series of normal tissues derived from different human organs. Scale bars: 200 μm. B Analysis of MYSM1 expression data from different tumor tissues and each corresponding adjacent normal tissue in the GEPIA database. C Analysis of MYSM1 mRNA levels in a variety of cell lines from different human tumors by qRT-PCR. The dark columns represent the cell lines with greater metastatic potential or a higher tumor grade. The dotted lines distinguish the cell lines on the basis of their different attributes (normal vs. tumor cells or cells with different migration potentials or grades). The data are shown as the means ± SDs (n = 3 independent experiments). [file 13046_2021_2106_MOESM11_ESM.jpg]

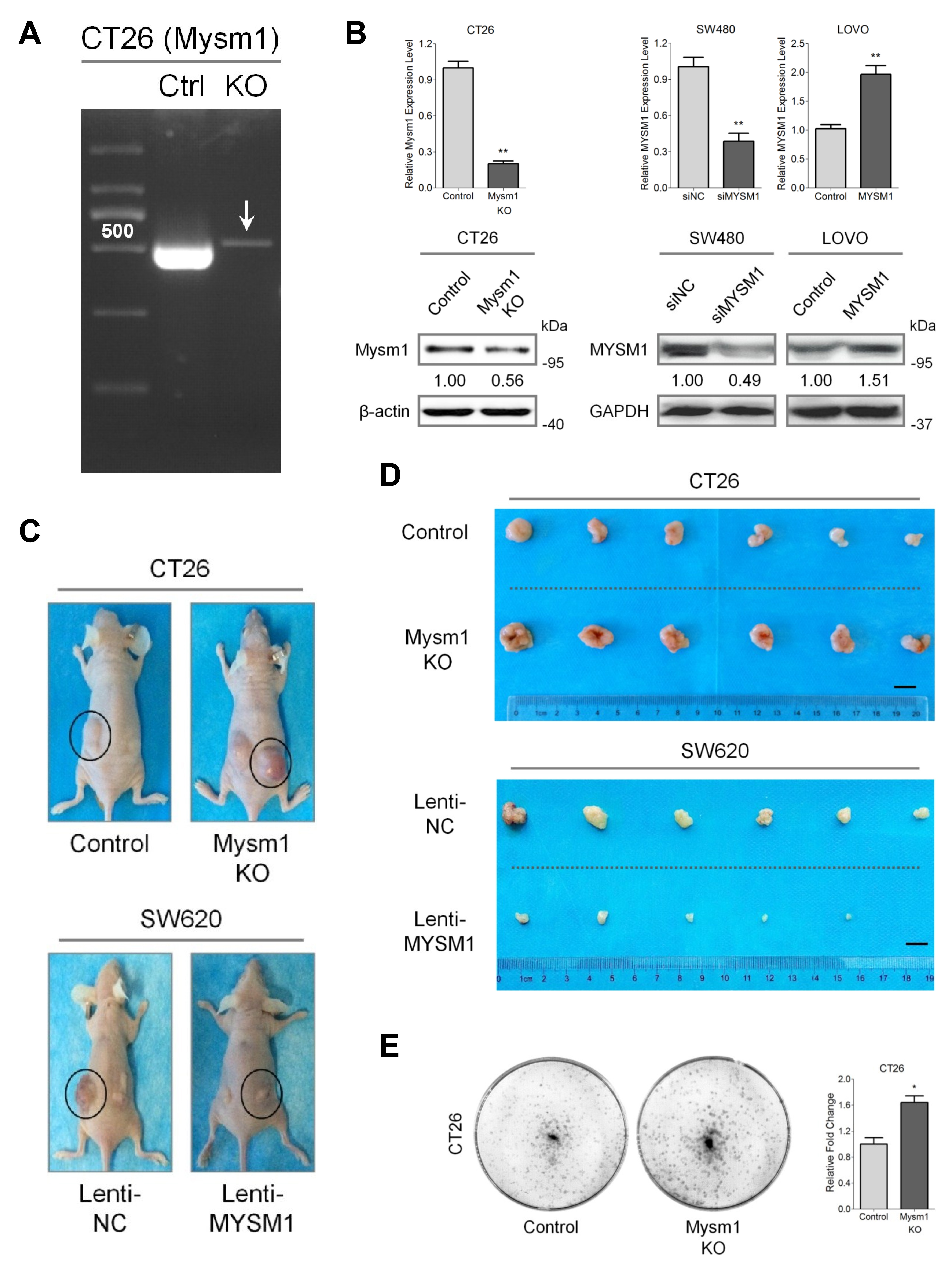

Supplement: Supplementary file 12 — Additional file 12: Figure S2. MYSM1 suppresses CRC cell proliferation in vitro and in vivo. A Efficiency of Mysm1 knockout by CRISPR-Cas9 in CT26 cells. Mysm1 expression fragments in the genome were detected by PCR. B Analysis of MYSM1 mRNA and protein levels in differently treated cells (CT26 cells, CRISPR-Cas9; SW480 cells, transient knockdown; and LOVO cells, transient overexpression) via qRT-PCR (upper) and western blot (lower). The data are presented as the means ± SDs (**P < 0.01, n = 3 independent experiments). C Representative photographs of tumor-bearing mice that received subcutaneous injections. D Neoplasms removed from the sacrificed mice are shown from large to small according to volume. Scale bars: 1 cm. E Colony formation analysis of engineered CT26 cells in vitro. The error bars indicate the SDs (*P < 0.05, n = 3 independent experiments). [file 13046_2021_2106_MOESM12_ESM.jpg]

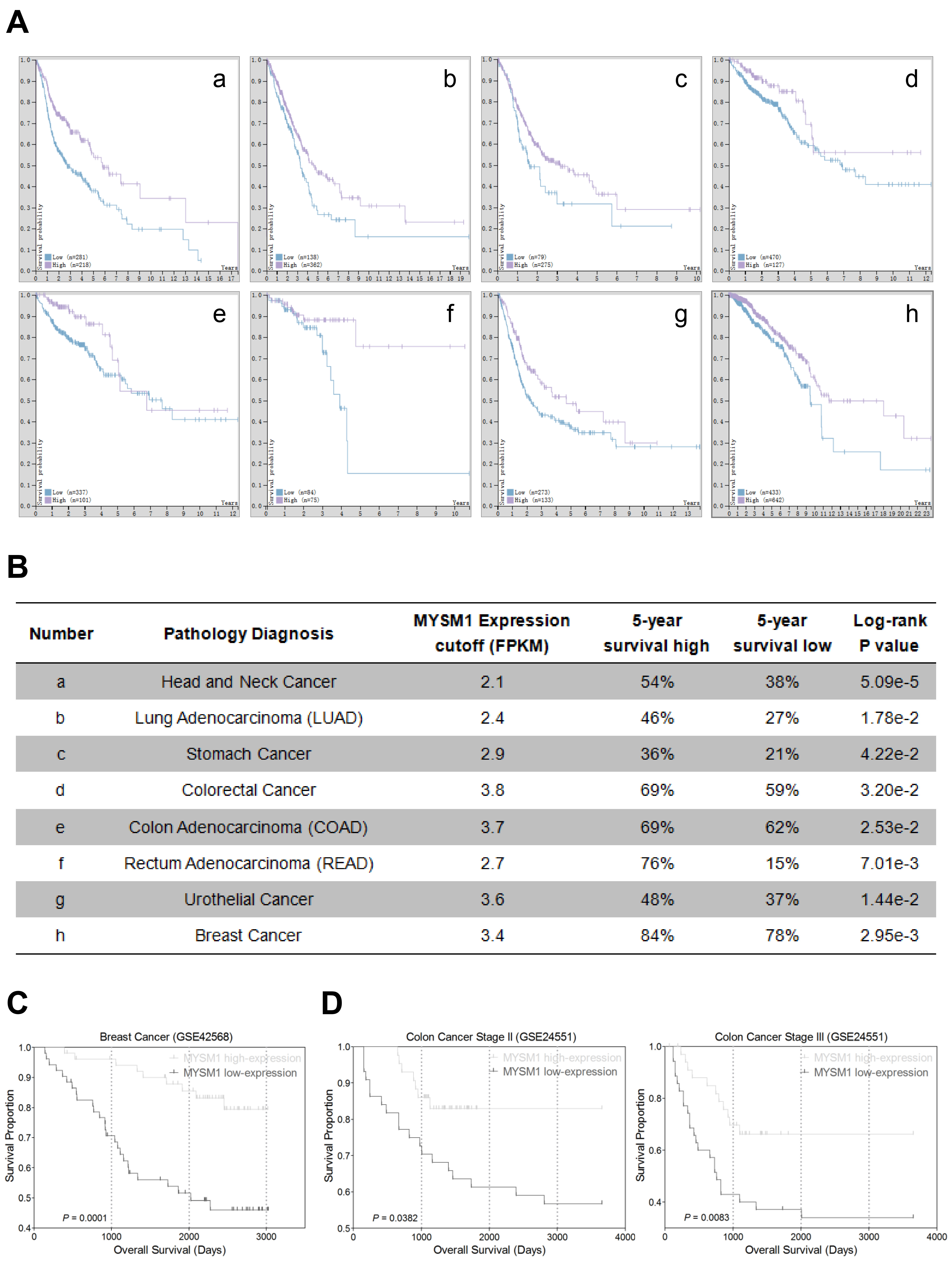

Supplement: Supplementary file 13 — Additional file 13: Figure S3. MYSM1 is a favorable biomarker in several tumor patients. A Kaplan-Meier survival curve analysis of patients divided by MYSM1 expression in different tumors based on data from The Human Protein Atlas. The P log-rank test was used to determine statistical significance. B Table summarizing the results of statistical analysis of the 5-year survival rate based on data from The Human Protein Atlas. C Kaplan-Meier survival curve of OS based on the MYSM1 expression levels in breast cancer patients from the GEO database (GSE42568). Statistical significance was analyzed by the P log-rank test. D Kaplan-Meier analysis of the OS of patients with different stages of CRC (stages II-III) (GSE24551) according to their MYSM1 expression levels. The P log-rank test was used to evaluate statistical significance. [file 13046_2021_2106_MOESM13_ESM.jpg]

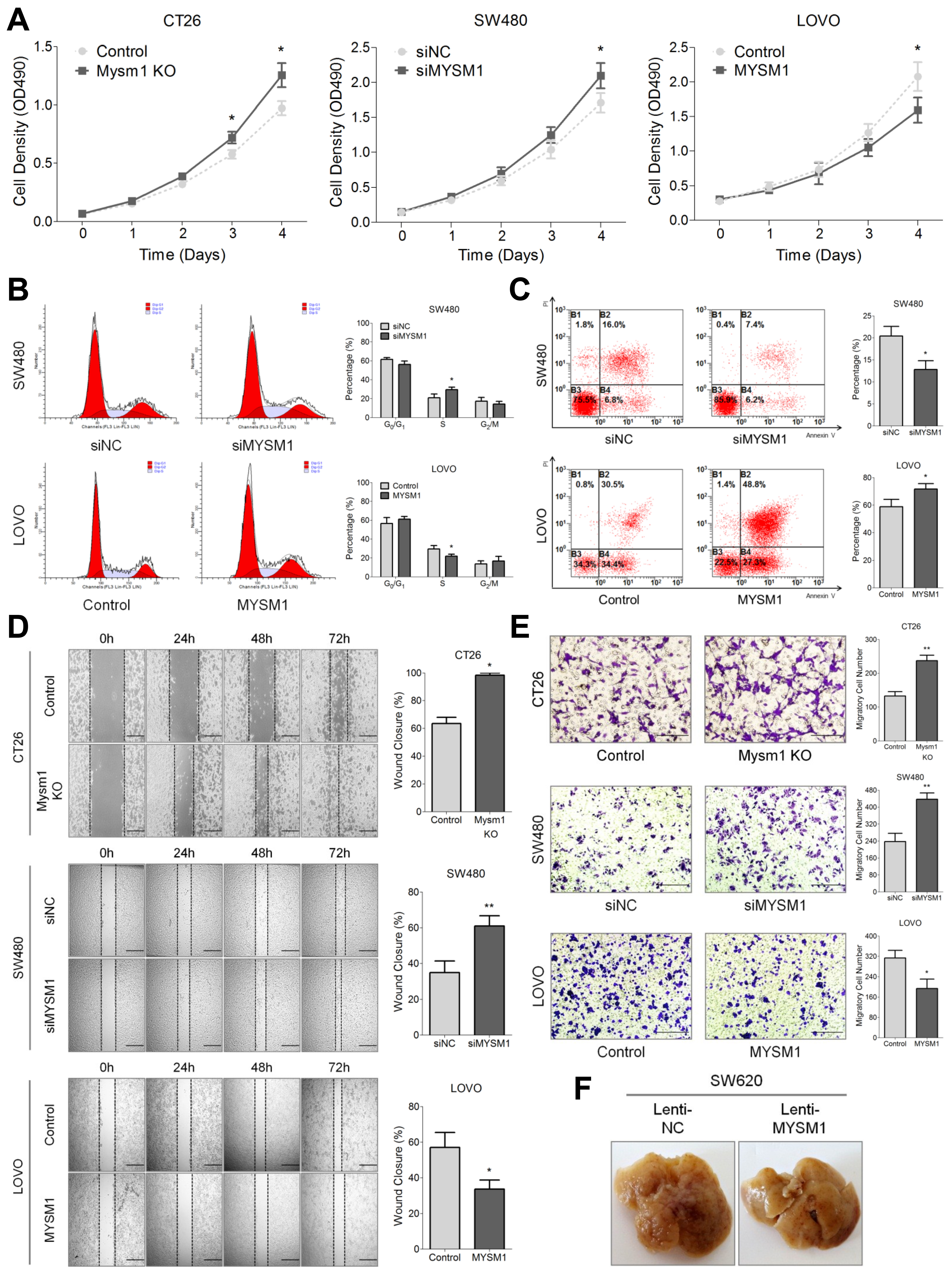

Supplement: Supplementary file 14 — Additional file 14: Figure S4. MYSM1 inhibits the proliferative and metastatic capacities of CRC cells in vitro. A Growth curve analysis of the proliferation of Mysm1-knockout (KO)/control CT26 cells, siMYSM1−/siNC-transfected SW480 cells and MYSM1-overexpressing/control LOVO cells. The error bars indicate the SDs (*P < 0.05, n = 6 independent experiments). B and C Analysis of the cell cycle (B) and apoptosis (C) in siMYSM1−/siNC-transfected SW480 cells (upper) and MYSM1-overexpressing/control LOVO cells (lower) by flow cytometry in vitro. D and E The metastatic capacity of Mysm1-KO/control CT26 cells, siMYSM1−/siNC-transfected SW480 cells and MYSM1-overexpressing/control LOVO cells was measured by wound healing (D) and Transwell (E) assays. Scale bars: 50 μm. The data in B-E are presented as the means ± SDs (*P < 0.05 and **P < 0.01, n = 3 independent experiments). F Representative graphs of typical lesions in the livers of athymic BALB/c mice that received tail vein injections of Lenti-MYSM1−/Lenti-NC-infected SW620 cells. [file 13046_2021_2106_MOESM14_ESM.jpg]

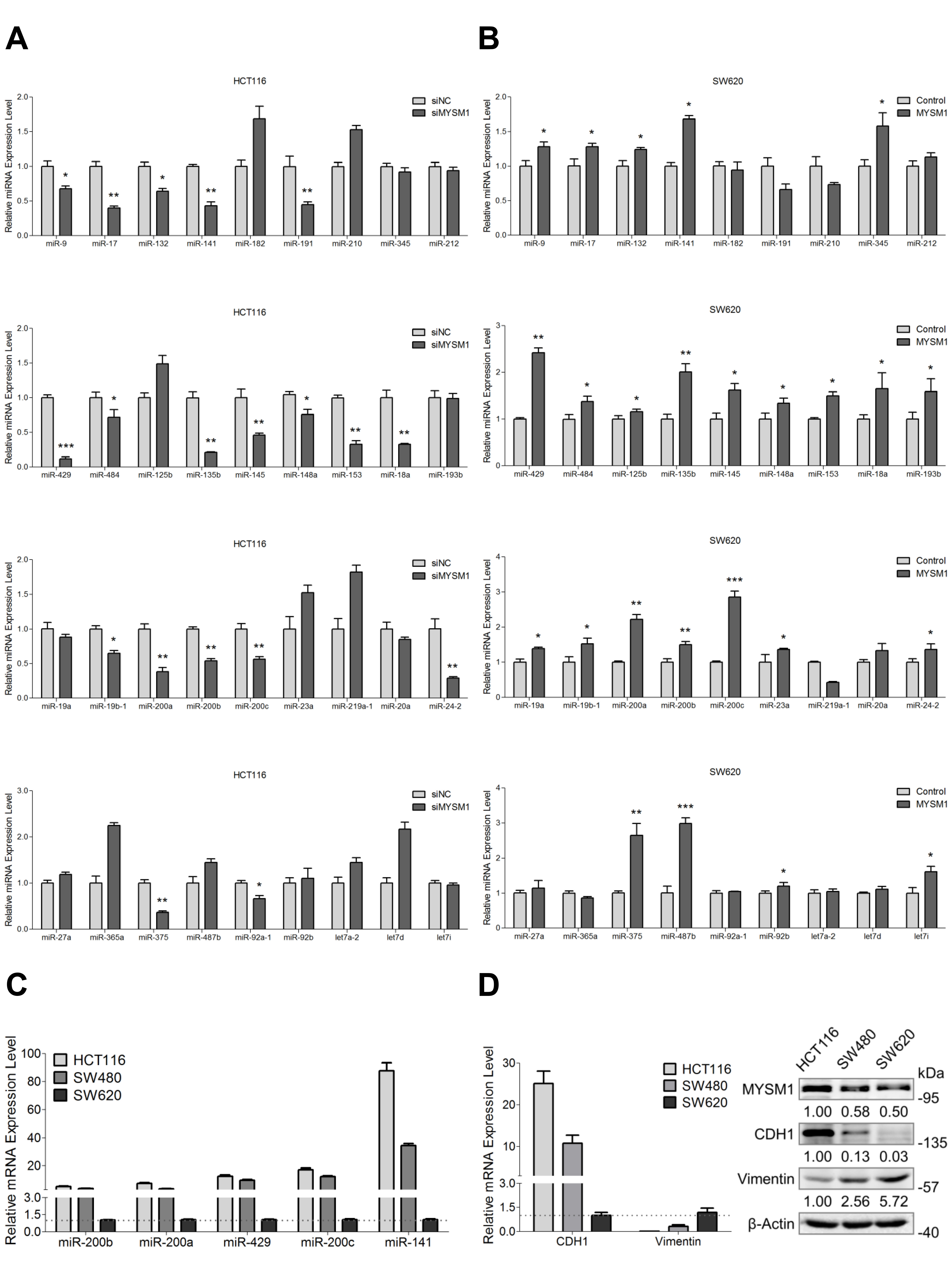

Supplement: Supplementary file 15 — Additional file 15: Figure S5. MYSM1 promotes the expression of miR-200 family members and inhibits the EMT process in CRC. A and B qRT-PCR analysis of the detailed alterations in the expression levels of 36 target miRNAs following MYSM1 downregulation in HCT116 cells (A) or MYSM1 upregulation in SW620 cells (B). The error bars represent the SDs (*P < 0.05, **P < 0.01 and ***P < 0.001, n = 3 independent experiments). C The mRNA levels of miR-200b, miR-200a, miR-429, miR-200c and miR-141 were measured by qRT-PCR in HCT116, SW480 and SW620 cells. The dotted line represents the normalized value of 1. The data are presented as the means ± SDs D The endogenous levels of MYSM1, CDH1 and vimentin in HCT116, SW480 and SW620 cells were examined by qRT-PCR (left) and western blot (right). The data are expressed as the means ± SDs. [file 13046_2021_2106_MOESM15_ESM.jpg]

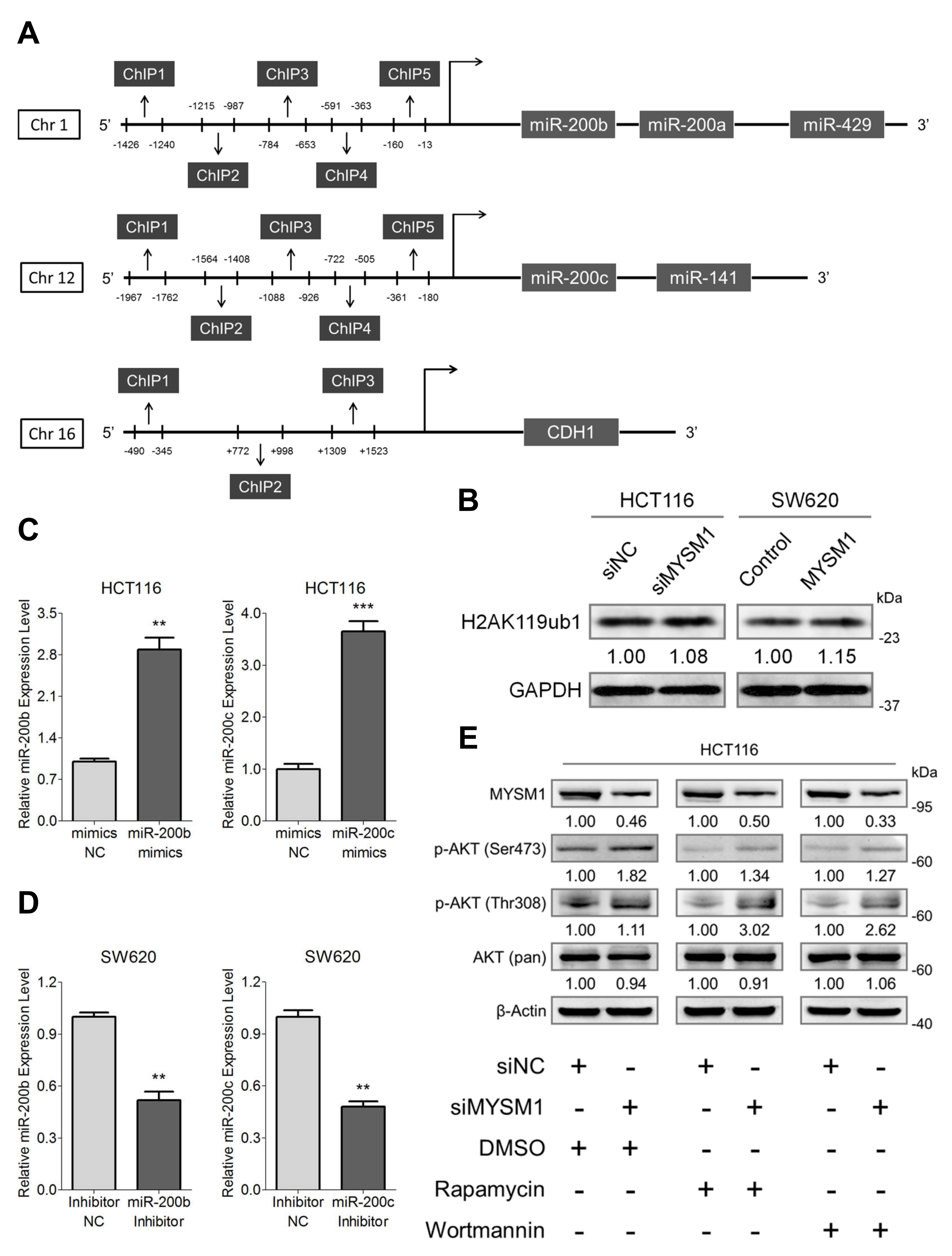

Supplement: Supplementary file 16 — Additional file 16: Figure S6. Pattern diagrams of the ChIP primers and restoration assay detection. A Locations and amplified fragments of the ChIP primers for the promoter regions of miR-200 family members and CDH1. The promoter regions of miR-200b cluster (chromosome 1, base pairs 0 ~ − 1500), miR-200c cluster (chromosome 12, base pairs 0 ~ − 2000) and CDH1 (chromosome 16, base pairs − 500 ~ + 1000) are shown. B The total protein expression of H2AK119ub1 was analyzed via western blot in HCT116 cells with MYSM1 knockdown and SW620 cells with MYSM1 overexpression. C and D qRT-PCR analysis of miR-200b and miR-200c levels in HCT116 (C) and SW620 (D) cells transfected with mimics/inhibitors compared with cells transfected with mimic/inhibitor NCs. The data are presented as the means ± SDs (**P < 0.01 and ***P < 0.001, n = 3 independent experiments). E Western blot analysis showing the rescuing effects of rapamycin (10 nmol/L) and wortmannin (100 nmol/L) on p-AKT (Ser473) and p-AKT (Thr308) in the context of MYSM1 knockdown-mediated PI3K/AKT signaling activation. [file 13046_2021_2106_MOESM16_ESM.jpg]

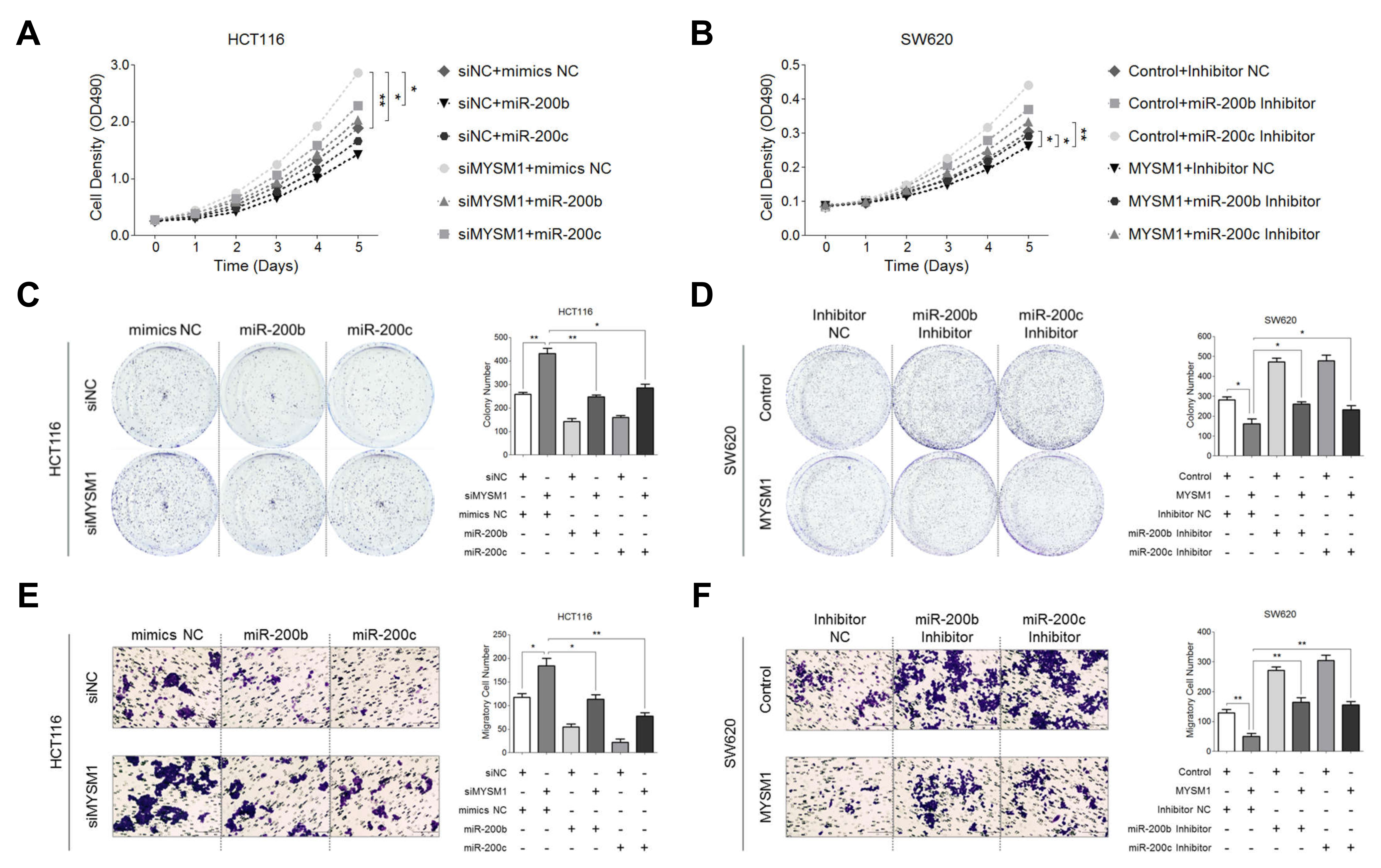

Supplement: Supplementary file 17 — Additional file 17: Figure S7. MYSM1 restrains the progression of EMT in CRC cells by inducing the activation of miR-200 family members. HCT116 cells were transiently transfected with siNC, siMYSM1, mimic NC, miR-200b or miR-200c mimic as indicated; SW620 cells were transiently transfected with the control, pMSCV-MYSM1 vector, inhibitor NC, miR-200b or miR-200c inhibitor as indicated. A-D The proliferation ability of the transfected HCT116 (left) and SW620 (right) cells was measured by growth curve (A and B) and colony formation (C and D) assays, respectively. E and F Transwell assays were used to measure migration in HCT116 (E) and SW620 (F) cells transfected with the indicated constructs. Scale bars: 50 μm. The data are shown as the means ± SDs (*P < 0.05 and **P < 0.01, n = 3 independent experiments). [file 13046_2021_2106_MOESM17_ESM.jpg]

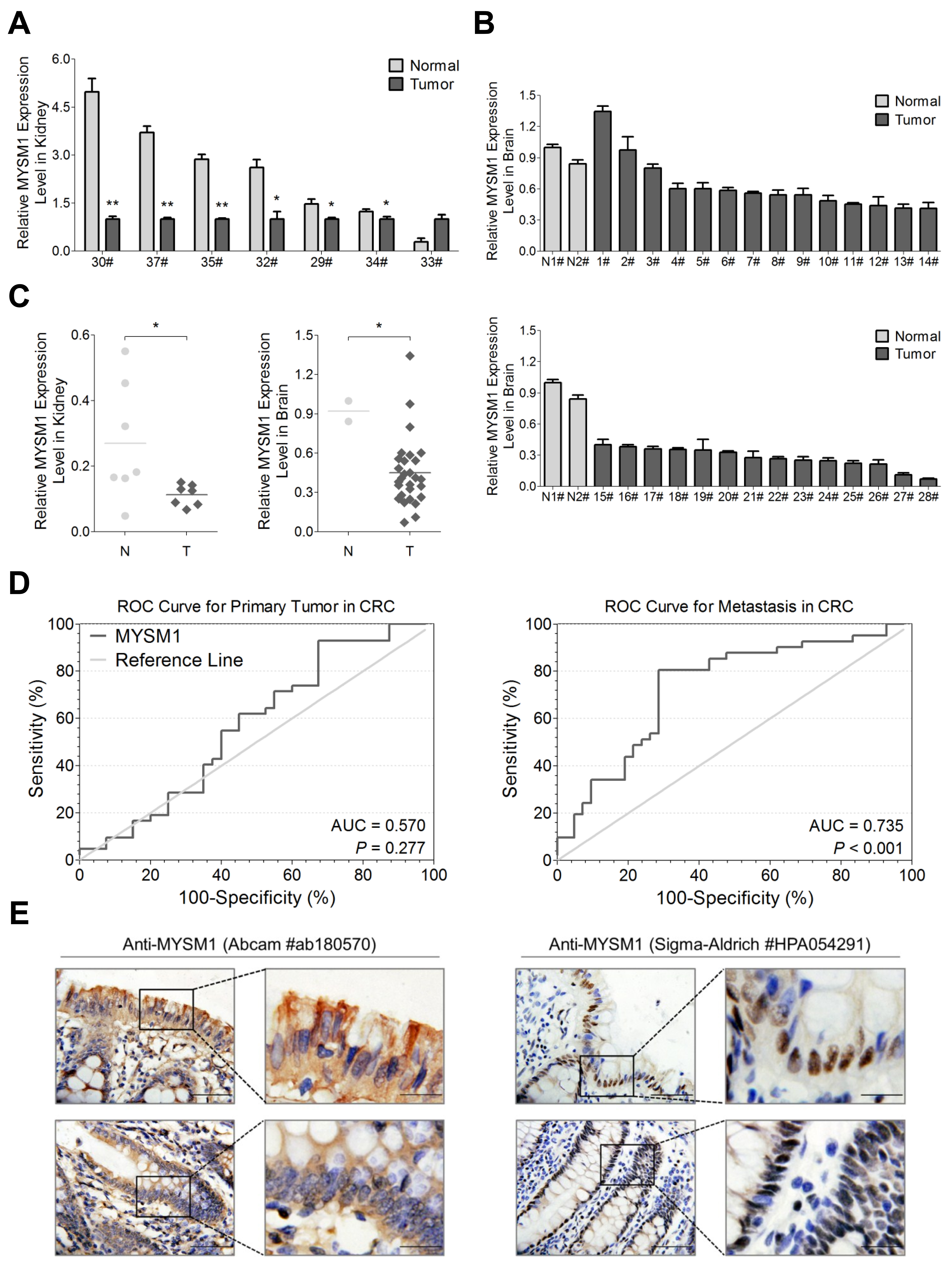

Supplement: Supplementary file 18 — Additional file 18: Figure S8. MYSM1 is significantly suppressed in tumor tissues and is located mainly in the nucleus rather than the cytoplasm. A qRT-PCR analysis of the MYSM1 mRNA levels in seven paired patient specimens with kidney tumors (T) and adjacent normal tissues (N). B qRT-PCR analysis of the MYSM1 mRNA levels in 30 unpaired patient specimens, including 28 gliomas (T) and two adjacent normal tissues (N). C Scatter plot showing the distribution of MYSM1 expression in normal and tumor tissues in the kidneys (left) and brain (right). The data (A-C) are presented as the means ± SDs (*P < 0.05 and **P < 0.01, n = 3 independent experiments). D ROC curve analysis was used to distinguish MYSM1 expression in primary tumors (left) and metastatic tumors (right) of CRC patients from that in tissues of healthy individuals. The data are shown as the AUC values. E Comparison of two MYSM1 antibodies (Abcam #ab180570, Sigma-Aldrich #HPA054291) and identification of MYSM1 localization by IHC. Scale bars: 200 μm (low power), 50 μm (high power). [file 13046_2021_2106_MOESM18_ESM.jpg]
